# Double-stranded RNA drives SARS-CoV-2 nucleocapsid protein to undergo phase separation at specific temperatures

**DOI:** 10.1093/nar/gkac596

**Published:** 2022-07-25

**Authors:** Christine A Roden, Yifan Dai, Catherine A Giannetti, Ian Seim, Myungwoon Lee, Rachel Sealfon, Grace A McLaughlin, Mark A Boerneke, Christiane Iserman, Samuel A Wey, Joanne L Ekena, Olga G Troyanskaya, Kevin M Weeks, Lingchong You, Ashutosh Chilkoti, Amy S Gladfelter

**Affiliations:** Department of Biology, University of North Carolina at Chapel Hill, Chapel Hill, NC 27599, USA; Lineberger Comprehensive Cancer Center, University of North Carolina at Chapel Hill, Chapel Hill, NC 27514, USA; Department of Biomedical Engineering, Duke University, Durham, NC 27708, USA; Department of Chemistry, University of North Carolina at Chapel Hill, Chapel Hill, NC 27514, USA; Department of Biology, University of North Carolina at Chapel Hill, Chapel Hill, NC 27599, USA; Curriculum in Bioinformatics and Computational Biology, University of North Carolina at Chapel Hill, Chapel Hill, NC 27599, USA; Department of Applied Physical Sciences, University of North Carolina at Chapel Hill, Chapel Hill, NC 27599, USA; Laboratory of Chemical Physics, National Institute of Diabetes and Digestive and Kidney Diseases, National Institutes of Health, Bethesda, MD 20892-0520, USA; Flatiron Institute, Simons Foundation, New York, NY 10010, USA; Department of Biology, University of North Carolina at Chapel Hill, Chapel Hill, NC 27599, USA; Department of Chemistry, University of North Carolina at Chapel Hill, Chapel Hill, NC 27514, USA; Department of Biology, University of North Carolina at Chapel Hill, Chapel Hill, NC 27599, USA; Department of Chemistry, University of North Carolina at Chapel Hill, Chapel Hill, NC 27514, USA; Department of Biology, University of North Carolina at Chapel Hill, Chapel Hill, NC 27599, USA; Flatiron Institute, Simons Foundation, New York, NY 10010, USA; Department of Computer Science, Princeton University, Princeton, NJ 08540, USA; Lewis-Sigler Institute for Integrative Genomics, Princeton University, Princeton, NJ 08540, USA; Department of Chemistry, University of North Carolina at Chapel Hill, Chapel Hill, NC 27514, USA; Department of Biomedical Engineering, Duke University, Durham, NC 27708, USA; Center for Genomic and Computational Biology, Duke University, Durham, NC 27708, USA; Department of Molecular Genetics and Microbiology, Duke University School of Medicine, Durham, NC 27708, USA; Department of Biomedical Engineering, Duke University, Durham, NC 27708, USA; Department of Biology, University of North Carolina at Chapel Hill, Chapel Hill, NC 27599, USA; Lineberger Comprehensive Cancer Center, University of North Carolina at Chapel Hill, Chapel Hill, NC 27514, USA

## Abstract

Nucleocapsid protein (N-protein) is required for multiple steps in betacoronaviruses replication. SARS-CoV-2-N-protein condenses with specific viral RNAs at particular temperatures making it a powerful model for deciphering RNA sequence specificity in condensates. We identify two separate and distinct double-stranded, RNA motifs (dsRNA stickers) that promote N-protein condensation. These dsRNA stickers are separately recognized by N-protein's two RNA binding domains (RBDs). RBD1 prefers structured RNA with sequences like the transcription-regulatory sequence (TRS). RBD2 prefers long stretches of dsRNA, independent of sequence. Thus, the two N-protein RBDs interact with distinct dsRNA stickers, and these interactions impart specific droplet physical properties that could support varied viral functions. Specifically, we find that addition of dsRNA lowers the condensation temperature dependent on RBD2 interactions and tunes translational repression. In contrast RBD1 sites are sequences critical for sub-genomic (sg) RNA generation and promote gRNA compression. The density of RBD1 binding motifs in proximity to TRS-L/B sequences is associated with levels of sub-genomic RNA generation. The switch to packaging is likely mediated by RBD1 interactions which generate particles that recapitulate the packaging unit of the virion. Thus, SARS-CoV-2 can achieve biochemical complexity, performing multiple functions in the same cytoplasm, with minimal protein components based on utilizing multiple distinct RNA motifs that control N-protein interactions.

## INTRODUCTION

Phase separation has long been described in polymer physics but only relatively recently is an appreciated mode of macromolecular self-assembly in cells that results in the formation of micron-scale droplets contributing to numerous cellular functions (1–3). While many of the mechanisms for protein-based condensation into droplets are known, the rules for partitioning specific nucleic acids are largely undefined. A model of ‘stickers and spacers’ describes many phase-separated coupled percolation systems (PSCP) (4,5) in which ‘stickers’ represent sites of interactions amongst polymers and ‘spacers’ are the intervening sequences between the association sites (6–8). The grammar of protein–protein interaction ‘stickers’ amongst disordered proteins and oligomerization domains is beginning to be established (6,9–15). How ‘stickers’ are encoded for RNA–protein or RNA–RNA interactions to promote condensates of specific identity and properties is far more mysterious (16).

Viruses present an opportunity to dissect interactions between proteins and nucleic acids that lead to liquid-like condensates because of their limited proteome that must engage with specific viral nucleic acids (i.e. viral genome). Indeed, proteins and nucleic acids from many different viruses have now been shown to undergo condensation in physiological conditions and form droplets in cells (17–23). Importantly, viral model systems involving one protein and one genomic nucleic acid (such as RNA), can reveal new principles for nucleic acid sequence- and structure-encoded phase separation by virtue of their compositional simplicity relative to multi-component condensates. We predict that viruses store information in their nucleic acid sequence and RNA structure to encode different condensate-dependent functions to achieve biochemical complexity, performing multiple roles in the same cell with few components. In this study, we manipulate RNA sequence and structure to decode RNA features that specify condensation of SARS-CoV-2 nucleocapsid (N-protein) and genomic RNA.

Although, the global COVID-19 pandemic motivated many studies of N-protein condensates, the specific role(s) of such assemblies in the viral replication cycle is still an open problem. The nucleocapsid protein (N-protein) is required for multiple viral functions (24). N-protein has many features associated with proteins that undergo phase separation including two RNA binding domains (RBD1 and RBD2) and additional intrinsically disordered motifs (25). Notably, N-protein displays lower critical solution temperature behavior (LCST), and RNA tunes the temperature at which N-protein forms droplets (26). N-protein forms droplets during infection (27), when expressed in cells and cell-free (9,26,28–36) with fragments of the viral RNA genome (26,29,33). N-protein condensates are dependent on salt (32–34), pH (34,37), and RNA sequence (26,29,32,33). Although RNA is required to induce N-protein demixing at physiological temperatures and ion conditions, the RNA sequence and structural preferences that govern N-protein interactions with RNA are unknown. Remarkably, RNAs of the same length but different sequence and structure do not equally drive N-protein condensation (26). This specificity indicates that N-protein condensation is encoded by sequence- and structure-specific interactions with RNA. Importantly, such differences raise the possibility that during infection, separate N-protein functions could occur in molecularly distinct droplets, whose identity is formed via RNA-components.

N-protein phase separation shows remarkable specificity for RNA sequence but the mechanism for N-protein's recognition of RNA is unknown. We previously showed that the first 1000 nucleotides of the SARS-CoV-2 genome (termed 5′end RNA) drive N-protein droplet assembly. In contrast, another RNA sequence of identical length surrounding the frameshifting element (FS) promoted solubilization of N-protein (26). A clue to these opposing effects came from the observation that these two RNAs exhibited differential crosslinking patterns with N-protein. Crosslinking between the 5′end RNA and N-protein preferentially occurred in specific single-stranded areas adjacent to structured elements. In contrast, crosslinking was uniformly distributed in the solubilizing FS sequence. We speculated that the differential crosslinking between condensation-promoting and solubilizing RNA could be used as a tool to identify N-protein preferences for particular RNA sequences, revealing how different modes of protein-RNA interactions influence condensates. Thus, we sought to uncover how N-protein recognizes RNA to promote the formation of liquid-like assemblies.

We show that the two RNA-binding domains in N-protein interact with distinct RNA-sequence and structure elements. This indicates N-protein has at least two distinct types of protein-RNA interaction ‘stickers’ that could provide multivalency for phase separation. RBD1 recognizes transcription-regulating sequence (TRS) and similar sequences in an RNA structure dependent manner. RBD2 specifically interacts with dsRNA, independent of sequence. The patterning and quality of these stickers can lead to emergent material properties of condensates. N-protein ‘RNA stickers’ can specify condensation temperature, RNA translation efficiency, sgmRNA generation, and genome condensation. Our work provides the first evidence of dsRNA/RBD interactions in specifying temperature-sensitive behavior in any phase separating system. Importantly, we identify how combinations of the two dsRNA stickers can pattern protein-RNA interactions to regulate condensation with important implications for betacoronavirus replication.

## MATERIALS AND METHODS

### Protein production

#### Recombinant protein expression and purification

For protein purification, full-length N-protein was tagged with an N-terminal 6-Histidine tag (pET30b-6xHis-TEV-Nucleocapsid, N-Y109A and N-RBD2-Del) were expressed in BL21 *Escherichia coli* (New England Biolabs). All steps of the purification after growth of bacteria were performed at 4°C. Cells were lysed in lysis buffer (1.5 M NaCl, 20 mM Phosphate buffer pH 7.5, 20 mM Imidazole, 10 mg/ml lysozyme, 1 tablet of Roche EDTA-free protease inhibitor cocktail Millipore Sigma 11873580001) and via sonication. The lysate was then clarified via centrifugation (SS34 rotor, 20 000 rpm 30 min) and the supernatant was incubated and passed over a HisPurTM Cobalt Resin (ThermoFisher Scientific 89965) in gravity columns. The resin was then washed with 4 × 10 CV wash buffer (1.5 M NaCl, 20 mM Phosphate buffer pH 7.5, 20 mM imidazole) and protein was eluted with 4 CV elution buffer (0.25 M NaCl, 20 mM phosphate buffer pH 7.5, 200 mM imidazole). The eluate was then dialyzed into fresh storage buffer (0.25 M NaCl, 20 mM phosphate buffer) and aliquots of protein were flash frozen and stored at –80°C. Protein was checked for purity by running an SDS-PAGE gel followed by Coomassie staining as well as checking the level of RNA contamination via Nanodrop and through running of a native agarose RNA gel. All experiments were performed with His-tagged N-protein. Whi3 was purified according to our established protocols (38,39).

### DNA sequences for RNA and protein constructs are in the Supplement

#### Dyeing of N-protein

N-protein was dyed by adding (3:1) Atto 488 NHS ester (Millipore Sigma 41698) to purified protein and incubating mix at 4°C for 1 h with rocking. Unbound dye was removed by overnight dialysis into protein storage buffer. For phase separation assays percent of dyed protein was adjusted to 10% of total by dilution with undyed protein.

#### RNA template design/production

Template predicted structure was designed using Vienna fold (http://rna.tbi.univie.ac.at). Sequences were generated via site directed mutagenesis using overlapping oligos (IDT). DNA sequences of tested RNA fragments are in the supplement.

#### In vitro transcription

RNA production was carried out according to our established protocols (Langdon *et al.*, 2018). Orf1ab templates were synthesized (IDT) and cloned into pJet (ThermoFisher Scientific K1231) using blunt end cloning. Directionality and sequence were confirmed using Sanger sequencing (GENEWIZ). Plasmid were linearized using PCR (iProof Bio-Rad 1725310). 5 μl of PCR product was loaded onto an agarose gel to determine size and purity. If the PCR product was pure then the sample was PCR purified (QIAGEN 28106) if the band was impure, it was gel purified (QIAGEN 28706) (PCR impurity was most often a problem for the ultrastructured mutants of principal site 2). 100 ng of gel or PCR purified DNA was used as a template for *in vitro* transcription (NEB E2040S) carried out according to the manufacturer's instructions with the addition of 0.1 μl of Cy3 (Sigma PA53026) or Cy5 (Sigma PA55026) labeled UTP to each reaction. Following incubation at 37°C for 18 h, *in vitro* transcription reactions were treated with DNAseI (NEB M0303L) according to the manufacturer's instructions. Following DNAse treatment, reactions were purified with 2.5 M LiCl precipitation. Purified RNA amounts were quantified using nanodrop and verified for purity and size using a denaturing agarose gel and Riboruler RNA ladder (Thermo Scientific SM183). Of note in an earlier version of this manuscript RNA molarity was calculated with a molecular weight of 499.5 (nucleotide triphosphate) rather than 321.5 (nucleotide monophosphate) meaning original reported values were inaccurate by a factor of ∼1.6.

#### Phase separation assays

For in vitro reconstitution phase separation experiments, 15 μl droplet buffer (20 mM Tris pH 7.5, 150 mM NaCl) was mixed with cy3 or cy5 labeled desired RNA and DEPC treated H20 (final volume 5 μl) and 5 μl protein in storage buffer was added at desired concentration. The mix was incubated in 384-well plates (Cellvis P384-1.5H-N) for 1–20 h at 37°C unless indicated otherwise. Droplets formed after short incubations of 20 min or less, however, they were initially smaller and matured into larger droplets during the overnight incubation step. Time to maturation varied based on the ratio of RNA to protein, concentration of RNA and protein and RNA sequence. Multiple conditions per mutant were tested with the most optimal conditions for differences selected for comparison. Imaging of droplets was done on a spinning disc confocal microscope (Nikon CSU-W1) with VC Plan Apo 100×/1.49 NA oil (Cargille Lab 16241) immersion objective and an sCMOS 85% QE 95B camera (Photometrics). Data shown are representative of three or more independent replicates, across two or more RNA preparations. Whenever possible multiple mutations were designed to disrupt the same class of feature in multiple sequence contexts.

### Comparison of droplet images to absorbance A280 reading in dilute phase

The mix was incubated in 384-well plates (Cellvis P384-1.5H-N) at 25, 30 or 37°C. Following imaging. 2 μl of dilute phase solution (taken from the top of the well) was nanodropped and absorbance *A*_280_ was recorded. Error bars indicate the A280 measurement from the three technical replicates. (Of note, concentrations below 3 μM N-protein did not give high enough A280 absorbance to generate reliable measurements.) *N* = 3 technical replicates.

#### Cell culture

HEK293T cells were originally obtained from ATCC. All cell lines were maintained in DMEM (Corning 10-013-CV) supplemented with 10% fetal bovine serum (Gibco). No antibiotics were used.

#### Plasmid transfection

Twenty four hours prior to transfection, confluent cells were split 1:5. Two hours prior to transfection, 500 μl of fresh media was added to 24-well plates. 500 ng of plasmid DNA for each Nucleocapsid GFP Spark (Sino biological VG40588-ACGLN) and the MSCV blast 1–1000 fragments was co-transfected using FUGENE HD. Transfections were then incubated for 24–48 h prior to imaging. *N* = 3 biological replicates.

#### Cell Imaging

Cells were imaged using a 40× air objective on a spinning disk confocal microscope (Nikon Ti-Eclipse, Yokogawa CSU-X1 spinning disk). Images were taken with a ANDOR camera. Representative cells are taken from *N* = 3 biological replicates.

#### Cell imaging quantification

Cells with puncta were cropped using FIJI. Experimenters were then blinded to conditions, and puncta were counted for each cell. Whole cell N:GFP signal was quantified using ImageTank (40).

#### Genome N-protein motif analysis

YRRRY motifs were counted throughout the NC_045512.2 reference genome (with overlapping motifs counted separately) and the motif counts in each 1000 bp window were plotted as a histogram. The density of double-stranded RNA was plotted using a kernel density estimation plot with smoothing parameter set to 100.

#### EMSA

65 ng/μl of the indicated RNA sequence was incubated with 0, 0.75, 1.5 or 2.2 μM Y109A mutant N-protein at 25 or 37°C for 1 h in the following buffer 10 mM HEPES pH 7.5, 50 μM EDTA, 10% glycerol, 1 mM DTT, 5 mM MgCl_2_, 0.1 mg/ml BSA, 2.5 μg yeast TRNA, 10 U RNAse inhibitor and loading dye. Samples were then loaded onto an 8% TBE gel and run at 100 V for 1 h at 4°C. Gels were then stained with SYBRgold (S11494) and imaged. Unbound RNA was quantified using ImageJ. *N* = 3 technical replicates.

#### Temperature dependent turbidity tests

The LCST behaviors of different phase separation systems were investigated on a Cary 300 temperature-dependent ultraviolet-visible spectroscopy equipped with a multicell thermoelectric temperature controller. The samples (4 μM of N-protein with 24 nM of RNAs) were mixed and prepared in a droplet buffer (20 mM Tris pH 7.5, 150 mM NaCl) at 4°C. Before the initiation of the heating process of the turbidity test, for the experiments shown in Figure 2A, the samples were incubated for 1 hour at 4°C; for the experiments shown in Figure 2B and C, the samples were incubated for 20 min at 4°C. A heating rate of 1°C/min was applied during the temperature ramp while the absorbance at λ  =  350 nm was recorded at every 0.33°C increment. Normalized turbidity was calculated by the absorbance at the lowest temperature point normalizes to the absorbance at the highest temperature point. *N* = 1 technical replicate.

#### Melting temperature of SL3

Melting temperatures were calculated using DINAMelt webserver. http://www.unafold.org/results2/twostate-fold/220509/163346/ Parameters for RNA at 37°C [Na^+^] = .15 M, [Mg^++^] = 0 M.

#### Phyre structure prediction/Pymol structure alignment

The following SARS-CoV-2 amino acid sequence was input into Phyre

TKKSAAEASKKPRQKRTATKAYNVTQAFGRRGPEQTQGNFGDQELIRQGTDYKHWPQIAQFAPSASAFFGMSRIGMEVTPSGTWLTYTGAIKLDDKDPNFKDQVILLNKHIDAYKTFP.

This sequence best matched with the crystal structure of the RBD2-dimerization domain of SARS-CoV-1 (41). The resulting structure prediction was aligned to the crystal structure of SARS-CoV-1 or MERS-CoV (42) using Pymol.

#### N-Protein beta sheet 2 conservation

N-protein amino acid sequences from MHV, OC43, MERS-CoV, SARS-CoV-1, and SARS-CoV-2 were taken from Uniprot and aligned using Clustal Omega.

#### RBD2/dimerization domain mutation frequency

Patient mutations from GSAID were downloaded on 7/14/2021 (2337992 total sequences for the following amino acids of N-protein.) for the following sequence.

TKKSAAEASKKPRQKRTATKAYNVTQAFGRRGPEQTQGNFGDQELIRQGTDYKHWPQIAQFAPSASAFFGMSRIGMEVTPSGTWLTYTGAIKLDDKDPNFKDQVILLNKHIDAYKTFP.

Fraction of samples with mutations for each amino acid was calculated by combining detected substitutions or indels and dividing by the total number of sequences. For highly mutated amino acids the most common mutation(s) were noted.

#### Mass photometry of purified N-protein

Mass photometry was performed according to established protocols (43). 10 μl of protein storage buffer (250 mM NaCl 20 mM phosphate buffer pH 7.5) was used to focus followed by addition of 10 μl of 40 nM N-protein in protein storage buffer (wildtype or RBD2-del) for a final protein concentration of 20 nM. Representative histograms were generated from 2 min movies reflective of the raw detected particle molecular weight in kDAs.

#### In vitro translation assay

Protocol was adapted from the method described by Tsang *et al.* (44) Briefly, 40nM of 5′UTR nano luciferase fusion RNA was incubated with either protein 0.3μM or 3.2 μM N-protein for 20-min at room temperature in PCR strip tubes (8μl total volume, final buffer conditions 140 mM NaCl, 4 mM phosphate buffer, 12 mM TRIS pH 7.5) as a control for basal luciferase RNA translation, N-protein storage buffer was added (250 mM NaCl 20 mM phosphate buffer pH 7.5). Following incubation, 5 μl rabbit reticulocyte lysate + Met + Leu (Promega L4960), was added to the protein/RNA mixture (or RNA and buffer) and the resulting mix was incubated at 30°C for 2 h. 2 μl of *in vitro* translation product was then mixed with 25 μl of nano luciferase assay reagents (Promega N205A). Light production was measured on a luminometer. Data depicted represents *N* = 3 replicates. Of note, similar translational repression was observed when we incubated RNA under droplet permissive conditions in plates (37°C, 1–2 h) however this was much less reproducible likely due to the difference in RNA partitioning in the well post incubation with N-protein.

#### Transmission electron microscopy (TEM) and quantification of RNP size distribution

For negative stained TEM images used to quantify the assemblies of RNP size distribution, 5 μl of 20 μM protein in 250 mM NaCl, 20mM phosphate buffer pH 7.5 and 5 μl of 80 nM RNA (FS RNA or 1000) in water were mixed in 15 μl of reaction buffer (150 mM NaCl, 20 mM Tris, pH 7.5). The final protein and RNA concentrations in the solution were 4 μM and 16 nM, respectively. For control measurements, protein without RNA and RNA without protein solutions were prepared. All mixture solutions were incubated at room temperature for overnight to measure negative stained TEM images.

Negatively stained samples were prepared on carbon film-coated grids supported by lacey carbon on 300 copper mesh (Electron microscopy Sciences). Grids were glow-discharged immediately before use. 8 μl aliquot of protein and RNA mixture solution was applied to the grid. After 2 min absorption to the carbon film, the solution was blotted and washed with 8 μl of water for 10 s, blotted, stained with 8 μl of 2% uranyl acetate for 10 s, blotted, and dried. Negative stained TEM images were obtained on a FEI Morgagni microscope.

Images were analyzed with ImageJ software (available at http://imagej.nih.gov/ij). Since the shape of the small RNP is not a sphere, two major and minor diameters of the elliptical shape of the RNP were measured, and the averaged values from two diameters were reported. Gaussian fitting of averaged diameter histogram was performed with Igor Pro 8.0.4.2 (WaveMetrics).

#### RNP-MaP probing of N-protein–RNA interactions

N-Protein and RNA mixtures were prepared as described in the ‘Phase Separation Assay’ section above and incubated for 1.5 h at 37°C. N-protein or N-protein Y109A–FS RNA mixtures were prepared in 80 nM RNA, 1μM protein (dilute state, 12.5× excess protein) RNA-only samples were also prepared as a control. After confirmation of phase separation by imaging mixtures were immediately subjected to RNP-MaP treatment as described (45), with modifications described below. Briefly, 200 μl of mixtures were added to 10.5 μl of 200 mM SDA (in DMSO) in wells of a 6-well plate and incubated in the dark for 10 min at 37°C. RNPs were crosslinked with 3 J/cm^2^ of 365 nm wavelength UV light. To digest unbound and crosslinked N-proteins, reactions were adjusted to 1.5% SDS, 20 mM EDTA, 200 mM NaCl and 40 mM Tris–HCl (pH 8.0) and incubated at 37°C for 10 min, heated to 95°C for 5 min, cooled on ice for 2 min, and warmed to 37°C for 2 min. Proteinase K was then added to 0.5 mg/ml and incubated for 1 h at 37°C, followed by 1 h at 55°C. RNA was purified with 1.8′ Mag-Bind TotalPure NGS SPRI beads (Omega Bio-tek), purified again (RNeasy MinElute columns, Qiagen), and eluted with 14 μl of nuclease-free water.

#### MaP reverse transcription

After SHAPE and RNP-MaP RNA modification and purification, MaP cDNA synthesis was performed using a revised protocol as described (Mustoe *et al.*, 2019). Briefly, 7 μl of purified modified RNA was mixed with 200 ng of random 9-mer primers and 20 nmol of dNTPs and incubated at 65°C for 10 min followed by 4°C for 2 min. 9 μl 2.2 2′-MaP buffer [1′ MaP buffer consists of 6 mM MnCl_2_, 1 M betaine, 50 mM Tris (pH 8.0), 75 mM KCl, 10 mM DTT] was added and the combined solution was incubated at 23°C for 2 min. 1 μl Superscript II Reverse Transcriptase (200 units, Invitrogen) was added and the reverse transcription (RT) reaction was performed according to the following temperature program: 25°C for 10 min, 42°C for 90 min, 10′ [50°C for 2 min, 42°C for 2 min], 72°C for 10 min. RT cDNA products were then purified (Illustra G-50 microspin columns, GE Healthcare).

#### Library preparation and sequencing

Double-stranded DNA (dsDNA) libraries for sequencing were prepared using the randomer Nextera workflow (46). Briefly, purified cDNA was added to an NEBNext second-strand synthesis reaction (NEB) at 16°C for 150 minutes. dsDNA products were purified and size-selected with SPRI beads at a 0.8 ratio. Nextera XT (Illumina) was used to construct libraries according to the manufacturer's protocol, followed by purification and size-selection with SPRI beads at a 0.65′ ratio. Library size distributions and purities were verified (2100 Bioanalyzer, Agilent) and sequenced using 2 × 300 paired-end sequencing on an Illumina MiSeq instrument (v3 chemistry).

#### Sequence alignment and mutation parsing

FASTQ files from sequencing runs were directly input into *ShapeMapper 2* software (47) for read alignment, mutation counting and SHAPE reactivity profile generation. The *–random-primer-len 9* option was used to mask RT primer sites with all other values set to defaults. For RNP-MaP library analysis, the protein:RNA mixture samples are passed as the *–modified* samples and no-protein control RNA samples as *–unmodified* samples. Median read depths of all SHAPE-MaP and RNP-MaP samples and controls were >50 000 and nucleotides with a read depth of <5000 were excluded from analysis.

Sub-genomic RNA abundance and recombination sites were taken from the following (48).

#### Secondary structure modeling

Secondary structure models were taken from our previous publication (26).

#### RNP-MaP reactivity analysis

A custom RNP-MaP analysis script (45) was used to calculate RNP-MaP ‘reactivity’ profiles from the *Shapemapper 2* ‘profile.txt’ output. RNP- MaP ‘reactivity’ is defined as the relative MaP mutation rate increase of the crosslinked protein–RNA sample as compared to the uncrosslinked (no protein control) sample. Nucleotides whose reactivities exceed reactivity thresholds are defined as ‘RNP-MaP sites’. RNP-MaP site densities were calculated over centered sliding 15-nt windows to identify RNA regions bound by N-protein. An RNP-MaP site density threshold of five sites per 15-nt window was used to identify ‘N-protein binding sites’ with boundaries defined by the RNP-MaP site nucleotides.

#### Dynamic light scattering

Dynamic light scattering (DLS) measurements were performed at 25°C using a Wyatt DynaPro temperature-controlled Plate Reader (Wyatt Technology, Santa Barbara, CA). Samples for the DLS system were prepared in the droplet buffer and filtered through 0.02 mm Whatman Anotop sterile syringe filters (GE Healthcare Life Sciences, Pittsburgh, PA) into a 96-well plate (Wyatt Technology, Santa Barbara, CA). Samples were incubated for 20 min at 25°C before testing. *N* = 10 acquisitions were taken, and the results presented represent the mean Rh of the sample.

#### Genome N-protein motif analysis

YYAAAY motifs were counted throughout the NC_045512.2 reference genome (with overlapping motifs counted separately) and the motif counts in each 1000 bp window were plotted as a histogram. The density of double-stranded RNA was plotted using a kernel density estimation plot with smoothing parameter set to 100. Viral genome RNA structure data was taken from (49).

## RESULTS

**Figure 1.**
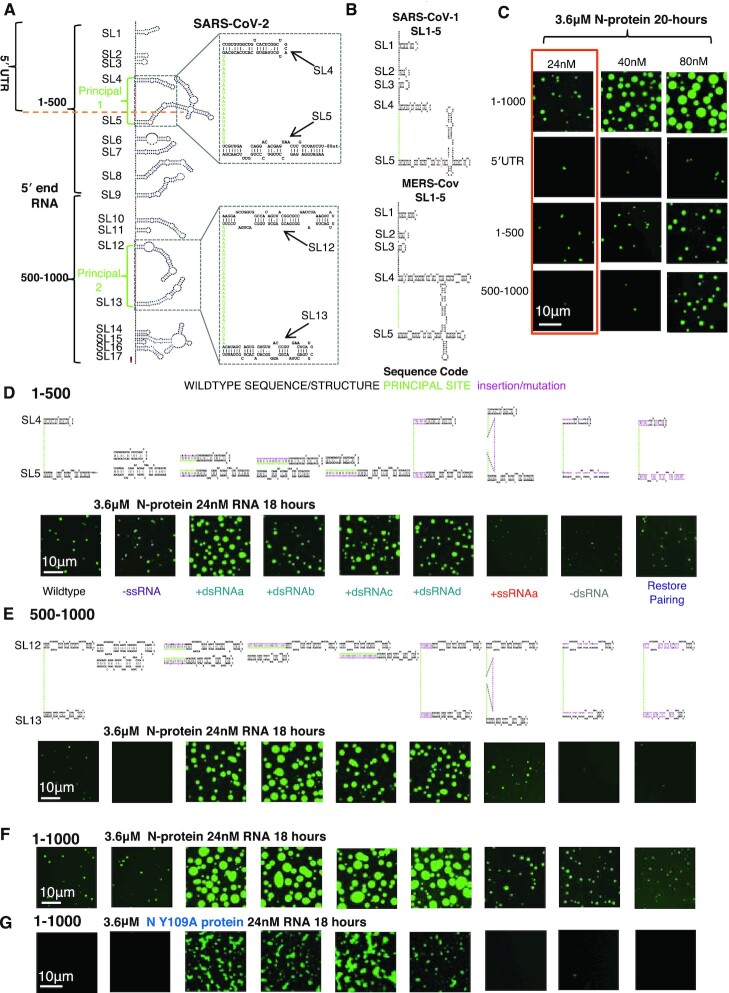
dsRNA-driven condensation is independent of RBD1. (**A**) SHAPE based structure model of the first 1000 nucleotides of the SARS-CoV-2 genome. Light green letters indicate locations of preferential N-protein crosslinking (principal sites). Brackets indicate the fragments; 5′UTR, 1–500 nt and 500–1000 nt. Stem-loops are numbered (SL). Inset indicates locations of structure manipulations for the rest of the figure. Specifically, mutations altered the region containing SL4 and 5 of principal 1 and/or SL12 and 13 principal 2. (**B**) Comparison of SL1-5 of SARS-CoV-1 and MERS-CoV (56). (**C**) Representative images from phase separation experiments with 3.6 μM recombinant N-protein (green) and the corresponding RNA sequence 1–1000, 5′UTR, 1–500 and 500–1000 for 24, 40 and 80 nM RNA. Orange box indicates selected condition for (D–F). (**D**) Mutation series in the 1–500 context depicting the predicted structure of mutants directed against SL4 and 5 and the intervening single stranded sequence of principal site 1 (light green letters). N-protein is depicted in green. Mutation classes are as follows -ssRNA (purple), +dsRNA (teal), +ssRNA (orange). -dsRNA (grey), Restore pairing (blue). (**E**) The equivalent mutation series (as in D) for 500–1000 context (principal site 2 in light green letters) depicting the predicted structure of mutants directed against SL12 and 13 or the intervening single stranded sequence of principal site 2. (**F**) Combination of mutations from (D) and (E) in the context of 1–1000. N-protein is depicted in green. (D–F) Deletion of the single stranded regions of the principal sites do not significantly impact condensation (-ssRNA). Addition of dsRNA (teal) (+dsRNAa-d) enhances N-protein condensation. Addition of single stranded RNA (+ssRNAa orange) coding for HA tag in the center of the principal sites leads to a mild enhancement of condensation. Unpairing principal site adjacent stem-loops (grey -dsRNA) on the 5′ side reduces condensation. Restoration of wildtype RNA structure (blue Restore pairing) but with a different sequence restores condensation to wildtype levels. (**G**) Only those mutations that lead to an addition of dsRNA (+dsRNAa-d), retain the ability to induce phase separation following Y109A mutation and destruction of N-protein RBD1. For all images, scale bar indicates 10 μm all experiments show representative images from at least three replicates and two independent batches of RNA.

We first sought to determine which RNA features promote N-protein condensation using an in vitro phase separation assay which we perform at physiological salt, protein and RNA concentrations in the absence of any artificial crowding agents. Previously, we identified two regions within the first 1000 nucleotides (nt) or 5′end of SARS-CoV-2 which preferentially crosslinked with recombinant N-protein at protein concentrations below those required for phase separation (principal sites). These principal sites are in single stranded sequences between two strongly structured (Figure 1A) and conserved (Figure 1B) stem-loops. Our goal here is to understand which features of this RNA sequence are the interaction sites of N-protein relevant for driving condensation. We hypothesize that these principal sites either act as ‘stickers’ that drive co-phase separation with N-protein or are ‘spacers’ adjacent to the functional stickers in the structured elements.

We first established a regime to be able to test each principal site independently and in combination in conditions which allowed us to see droplet size and/or morphology change following mutation. Principal site 1 is in the 5′UTR (nt:1–267 above orange dashed line) (Figure 1A). Given the observation that 5′UTR and smaller fragments can induce N-protein phase separation (29,33), we first asked what segments of the first 1000 nt of the 5′end were sufficient to promote condensation *in vitro*? To this end, we tested 1–1000 nt, 1–267 nt (the 5′UTR), 1–500 nt and 500–1000 nt fragments at either 24, 40 or 80 nM RNA and 3.6 μM protein. All tested fragments could drive N-protein condensation however some fragments drove condensation more readily (1–500 or 1–1000) (Figure 1C). We selected 3.6 μM N-protein and 24 nM RNA for subsequent experiments (orange box), which resulted in medium sized droplets for 1–1000 nt and 1–500 nt. Medium sized droplets allowed us to see reduction or enhancement of condensation following mutation providing a set of conditions to examine RNA features relevant to droplet formation.

### dsRNA promotes N-protein condensation

We predicted if N-protein recognizes ssRNA, altering the ssRNA content between stem-loops (increasing or decreasing) should alter N-protein binding to principal sites and in turn condensation. Alternatively, if adjacent dsRNA mediates N-protein recognition of principal sites, we predict that changing the length of stem-loops will alter N-protein binding and condensation. Thus, we designed a series of mutations to independently disrupt single stranded and double-stranded RNA in or adjacent to the principal sites.

We disrupted principal site 1 in 1–500 nt (Figure 1D), principal site 2 in 500–1000 nt (Figure 1E), or both principal sites in 1–1000 nt (Figure 1F). To test the importance of the single-stranded, principal site sequence alone, we first deleted the single stranded sequence (–ssRNA). We observed that in any of the tested sequence contexts (Figure 1D–F) deletion of the ssRNA principal site did not significantly alter condensation relative to wild-type. This shows that the ssRNA is not required for N-protein droplet formation. Instead, N-protein binding to dsRNA may drive condensation.

We next sought to address the role of the conserved structured RNA (Figure 1B) located adjacent to the principal sites. To do this, we converted the single-stranded, principal site sequence to dsRNA (preserving the total RNA length, by recoding the sequence 5′ and 3′ to the stem-loops to pair with the principal site (+dsRNAa). Strikingly, this type of mutation resulted in enhanced condensation in all three sequence contexts (Figure 1D–F), with much larger droplets forming more quickly in identical protein and RNA concentrations. We next sought to induce the formation of additional double-stranded RNA in a different way. Thus, we converted the single stranded principal site region to double-stranded RNA by forcing the single stranded region to base pair by adding complementary sequences. We did this either 5′ of the first hairpin (+dsRNAb) or 3′ of the second hairpin (+dsRNAc) which flanked the single stranded principal sites. In all three sequence contexts, this type of mutation again enhanced the rate of formation and volume fraction of condensed material (Figure 1D–F). Thus, using six different mutant contexts, we see dsRNA promotes condensate formation.

To examine if dsRNA addition could be additive, we also tested individually +dsRNAb and +dsRNAc on a single principal site (either 1 or 2) in the context of 1–1000 nt. We observed that mutations which affect principal site 2 were better able to enhance condensation compared to those which effect principal site 1 (Supplementary Figure S1A). This is likely due to dsRNA length differences. For example, mutated principal site 1 adding 44 nt of dsRNA (22 nt of additional RNA sequence) was less efficient at driving assemblies than principal two adding 62 nt of dsRNA (31 nt of additional RNA sequence) (Supplementary Figure S1A). Further, the combination of the mutations did not enhance droplet formation much more than those only altering principal site 2 which indicates there may be a threshold to the enhancement (Supplementary Figure S1A). These data suggest that increasing dsRNA content, up to a certain threshold, can accelerate and promote a larger volume of condensed N-protein.

So far, all tested mutations which enhanced structure also destroyed the single stranded principal site by converting it to dsRNA. Therefore, we next asked whether addition of 10 paired nucleotides (20 nt per stem-loop) of dsRNA at the base of the principal site flanking stem-loops would also promote condensation (+dsRNAd). This mutant would preserve the ssRNA of the principal site while creating additional structure. We observed that these extended stem-loops indeed also enhanced condensation relative to wildtype in all three sequence contexts (Figure 1D–F). A caveat to interpreting these results however is that 3/4 of the classes of +dsRNA (+dsRNAb–d) mutant RNA increases the length by 22–80 nt) and RNA length has been shown to modulate the ability of N-protein to form condensates (26).

We sought to disentangle the effects of length addition to the dsRNA constructs by expanding the single stranded region by inserting exogenous sequence (+ssRNAa) (27 nt coding for Hemagglutinin (HA)) in the center of the single stranded principal sites. Two different ssRNA sequences were independently inserted into principal site 2 (Supplementary Figure S1B). We observed in all three sequence contexts, addition of HA RNA sequence resulted in negligible enhancement of condensation (Figure 1D–F). These RNA length controls all resulted in negligible levels of enhancement (Supplementary Figure S1B). Taken together, these results suggest that dsRNA addition enhances N-protein condensation in contrast to ssRNA addition and the enhancement by additional dsRNA cannot be explained simply by increased RNA length.

Next, we sought to determine whether the sequence and/or structure of the stem–loops flanking the principal sites were important. Therefore, we unpaired the principal site flanking stem-loops by making mutations (–dsRNA) on the 5′ side. We observed that in all three contexts -dsRNA resulted in a reduction (1–500) or loss (500–1000, 1–1000) of condensation relative to wildtype (Figure 1D–F). To rescue the -dsRNA mutant we made compensatory mutations on the 3′ side of the stem-loop to restore the structure (Restore pairing). The Restore pairing mutant resembled wild-type levels of condensation in all sequence contexts. Thus, we concluded that reducing dsRNA generally limits phase separation and the specific primary sequence of the stem-loops does not play a significant role.

To assess if addition of dsRNA was sensitive to the relative stoichiometry of RNA and protein, we tested wildtype and +dsRNAa in the context of 1–1000 in a small phase diagram. +dsRNAa was chosen as it is the same exact length as wildtype but produced more and larger droplets at 3.6 μM N-protein and 24 nM RNA. We observed that relative to wildtype (Supplementary Figure S1C), +dsRNAa (Supplementary Figure S1D and E) consistently produced more condensates at 3 μM N-protein (Supplementary Figure S1C and D) indicating this enhancement is reproducible in multiple regimes. However, differences were observed at 1 μM N-protein with only some conditions promoting more condensate formation, indicating a shifted phase boundary for the mutant. (Supplementary Figure S1C and D). We further confirmed that N-protein recruitment to droplets was higher for +dsRNAa by measuring the absorbance of the dilute phase at 280 nm (*A*_280_) (Supplementary Figure S1E). In all three tested RNA concentrations at 3 μM N-protein mutant RNA addition resulted in significantly lower 280 signal indicative of higher levels of droplet recruitment (Supplementary Figure S1E). Thus, dsRNA-mediated enhancement of condensation appears to be consistent across different RNA and protein concentrations or ratios.

### dsRNA-driven condensation is independent of RBD1

We next determined which RNA binding domain of N-protein mediates the dsRNA-based condensation enhancement. N-protein has two distinct RNA-binding domains; located in the N-terminal domain (NTD) RBD1 is structured (50) and in the C-terminal domain (CTD) RBD2 is a lysine-rich IDR (51). The single point mutant Y109A in RBD1 blocked droplet formation with 5′end RNA (1–1000) (26) and resulted in a 2000-fold reduction in affinity for RNA (50). Y109A mutant N-protein was incubated with the panel of mutant RNAs in the context of 1–1000. Only those mutations which resulted in more dsRNA could promote condensate assembly (Figure 1G). Notably, the droplets that form with these more structured RNAs and Y109A are smaller and flocculated (different morphology) suggesting key aspects of the material properties of droplets are lost with the loss of RBD1 activity. Thus, +dsRNA can promote phase separation independent of a functional RBD1 suggesting +dsRNA works instead through interactions with RBD2.

We sought to test whether the condensation-promoting mutations in the RNA sequences were specific to N-protein or generalizable to any RNA-driven phase separating system. To this end, we tested all mutations in the 1–1000 context with recombinant Whi3 protein. Whi3 has previously been shown to undergo sequence-specific RNA-dependent phase separation (38,39). We observed no obvious difference between any of the mutant RNAs and the wildtype 1–1000 nt sequence with condensing Whi3 protein (Supplementary Figure S1F). This indicates that the mutations are acting specifically through alteration of N-protein/RNA interaction and not a general, non-specific RNA:protein interaction or trans RNA:RNA interaction. Taken together, addition of dsRNA enhances the phase separation of N-protein specifically, and this enhancement is independent of RBD1 and requires structure but not the specific RNA primary sequence.

### RNA structure mutants accelerate droplet formation in cells and in solution

Next, we sought to confirm our observations regarding RNA sequence/structure-mediated N-protein droplet formation in cells to see if the sequences behave similarly in the more complex and crowded cellular environment. To this end, we first needed to control for the reported translational repressive effects (52,53) of non-structural protein 1 (NSP1) which was encoded in the 5′ end 1–1000 fragment. Thus, we designed a mutation in the start codon of NSP1 (Start Mutant) which would preserve the structure of SL5 but block NSP1 translation (Supplementary Figure S2A). We then confirmed that the Start Mutant yielded similar levels of droplets as wild-type (Supplementary Figure S2B). It was unnecessary to also mutate the NSP1 start codon of our structure mutant of the same length (+dsRNAa) as this mutation also resulted in premature stop codons in NSP1 protein (Supplementary Figure S2C). Thus, we cloned wildtype 1–1000, Start Mutant, and +dsRNAa into a mammalian expression vector and co-transfected these plasmids with a plasmid driving N-protein:GFP in HEK293T cells (Supplementary Figure S2D).

To determine if dsRNA addition altered condensates in cells, we imaged cells co-transfected with specific RNAs and protein. We observed that at early timepoints (24 hours) +dsRNAa resulted in a significant increase in the number of puncta (4–5 per cell) per square micron in cells compared to Wildtype or Start Mutant control (2–3 per cell) (Supplementary Figure S2E and F). However, this difference was reduced at 48 hours. Further, there was no significant difference in the mean fluorescence of N:GFP between the compared cells (Supplementary Figure S2G) so differences could not be explained by N-protein expression levels (26). Collectively, these results suggest that dsRNA addition accelerates N-protein droplet formation in cells.

The apparent acceleration in droplet formation time prompted us to examine differences in timing with the in vitro system for mutants in all 3 sequence contexts (shorter incubation time (2 hours) than shown in Figure 1D–F (18 h). Consistent with the structure mutants accelerating N-protein droplet formation in cells, the mutants which result in more and larger droplets at 18 hours had vastly more pronounced differences at 2 h indicating that these structure mutants also accelerate droplet formation cell free (Supplementary Figure S2H). Similar results as for 1–1000 (Supplementary Figure S2H) were obtained for both the 1–500 and 500–1000 contexts (Supplementary Figure S2I and J). Collectively, these data suggest that addition of dsRNA accelerates N-protein phase separation with target RNAs both cell free and in cells.

### Addition of dsRNA alters material properties

We next asked if the addition of dsRNA alters the material properties of the resulting droplets. In our previous work, we found that different RNA sequences could lead to N-protein droplets with distinct physical properties. This feature may be relevant for generating immiscible droplet populations (16). In these previous experiments that generated different droplets, RNA sequences were different in length and structure. We decided to employ the +dsRNAa RNA to determine how minor alterations in RNA sequence and structure impact material properties in RNAs of the same length. To this end we tested wild-type 5′end RNA 1–1000 and +dsRNAa using 40K and 10K dextrans to examine droplet pore size. We performed these experiments at different RNA and protein ratios and concentrations than those in Figure 1F to allow for the formation of sufficiently large droplets in the wild-type context (Supplementary Figure S1C–E) at an earlier timepoint. We observed that droplets formed from all 3 sequences excluded 40K Dextrans following 0.5 hours of incubation (Supplementary Figure S3A) and were largely permeable to 10K dextrans (Supplementary Figure S3B) with +dsRNAa having weak but significant levels of exclusion of 10K dextrans (Supplementary Figure S3C and D). Consistent with previous results, +dsRNAa also resulted in significantly larger droplets (Supplementary Supplementary Figure S3E) which have higher Atto488 fluorescence (N-protein signal) (Supplementary Figure S3F) suggestive of more N-protein recruitment to droplets. Wildtype and +dsRNAa resulted in droplets with similar levels of circularity (Supplementary Figure S3G). Collectively, these results suggests that the altered dsRNA content can change droplet porosity possibly through additional N-protein recruitment to droplets.

### Increasing dsRNA content lowers LCST independent of total RNA length/sequence

N-protein will condense in the absence of RNA at high temperatures, but addition of RNA lowers the temperature at which droplets emerge. Thus N-protein displays lower critical solution temperature (LCST) behavior and RNA tunes this property (26). Notably, the addition of RNA lowers the LCST to physiological body temperatures and thus may relate to N-protein condensation in mammalian cells. It is unclear how RNA sequences and structures specify the temperature at which N-protein demixing occurs and which RNA binding domains are involved. Thus, we next sought to use the RNA sticker mutants identified in Figure 1 to assess the impact of RNA sequence on N-protein LCST behavior.

To determine how LCST behavior is encoded by RNA sequence and structure we first confirmed N-protein condensation temperature changed as a result of the co-condensing RNA (three different RNA sequences). To this end, we used a temperature-dependent ultraviolet-visible spectroscopy assay to map the saturation temperature, read out as turbidity to test if this assay could be used as a proxy for phase separation. We examined the following conditions: N-protein alone, N-protein + an RNA which does not drive condensation (Frameshifting region RNA (FS)(26), N-protein + 5′end RNA (1–1000nt) which drove condensation, or N-protein + Nucleocapsid RNA (drives condensation but is a longer sequence then 5′end).

**Figure 2.**
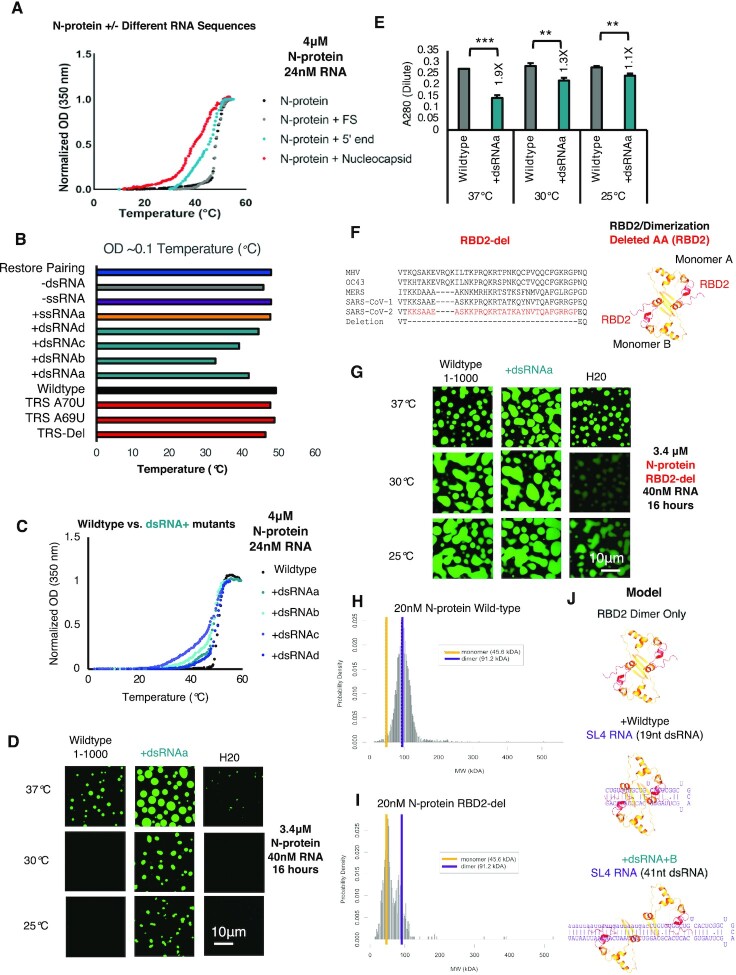
RNA sequence and structure encodes N-protein LCST behavior via RBD2. (**A**) Temperature dependent turbidity tests of N-protein alone (Black), N-protein with Frameshifting region RNA (FS) (Gray), and N-protein with 5′end RNA (1–1000nt) (Blue) and N-protein with Nucleocapsid RNA (Red). Addition of droplet forming RNAs, 5′end 1–1000nt and Nucleocapsid RNA to N-protein, lowers the transition temperature but solubilizing RNA (FS) does not. (**B**) Transition temperature comparison (repeat of the experiment shown in (**A**) of wildtype 5′end or 11 mutants in the context of 1–1000nt. Bar length indicates the temperature in°Celsius at which the turbidity of the solution reaches ∼0.1. Only those mutants which alter the dsRNA content (teal + dsRNA), lower the temperature at which OD reaches ∼0.1 indicative of increased solution turbidity. (**C**) Temperature dependent turbidity tests for N-protein plus wildtype 5′end RNA as well as the four more structured mutants (+dsRNA) which lower the transition temperature. (**D**) Validation of the turbidity assay using droplet imaging (Figure 2B and C). 3.4μM Wildtype N-protein was mixed with either 40nM of wildtype 5′end 1–1000 RNA, +dsRNAa (RBD1 independent Figure 1G) or water only added control (H20) and incubated at the indicated temperature 37, 30, or 25°C for a period of 20 hours prior to imaging. Consistent with previous results, +dsRNAa increases droplet size relative to wildtype at 37°C (Figure 1F) & induces condensation at lower temperatures. (**E**) A280 measurement of remaining N-protein in the dilute phase for (D). At all temperatures, +dsRNAa lowers A280 measurements relative to wildtype. Error bars mark standard deviation for the three replicates and * indicate significance students *t* test (*** *P* < 0.001, ** *P* < 0.01, *P < 0.05, ns not significant) with brackets showing comparison for the indicated statistical test. (**F**) Protein sequence conservation of N-protein RBD2 and structure model of the RBD2 dimerization domain for SARS-CoV-2 (red sequences/red ribbon) indicate the location of the deletion in the primary sequence tested in (G). (**G**) RBD2/Dimerization domain is required for proper N-protein LCST behavior at indicated temperature range. 3.4 μM of N-protein RBD-del (green) was mixed with 25nM of either wildtype 1–1000, +dsRNAa, or water only control and incubated at the indicated temperatures for 16 h. Droplet formation was observed in all conditions although RNA dependence was more evident at lower protein concentrations (Supplementary Figure S4G-H). (H, I) Mass photometry histograms showing the molecular weight (MW) distribution of detected particles for wild-type N-protein (**H**) or RBD-del N-protein (**I**). (H) Wildtype N-protein is a stable dimer in solution (250 mM NaCl pH 7.5 20 mM phosphate buffer 20 nM N-protein) but RBD2-del is mostly a monomer (I). (**J**) Model of N-protein RBD2/Dimerization domain interactions with dsRNA. Binding of the two RBD2s of the two monomers of N-protein to dsRNA facilitates dimerization dissociation with temperature facilitating dissociation for shorter stem-loops. For all images scale bar indicates 10 μm all experiments show representative images from at least 3 replicates.

Consistent with previous results, (26) we observed that N-protein + FS (which does not drive phase separation) and N-protein alone underwent phase separation at the same high temperature (Figure 2A) of ∼46°C. In contrast, the two condensation-promoting RNAs both lowered temperature, with 3′end Nucleocapsid RNA conferring a lower temperature then 5′end. The turbidity curves differ in shape depending on the specific RNAs such that condensation-promoting RNAs display a more gradual turbidity increase. While different RNAs promoted distinct LCST behavior, this could be due to sequence and/or length-dependent effects. Thus, we could use temperature-dependent ultraviolet-visible spectroscopy to determine how subtle variations in RNA sequence alter N-protein condensation temperature.

We wanted to identify the RNA and protein features which were responsible for conferring N-protein LCST independent of RNA length (Nucleocapsid RNA is longer then 5′end). To disentangle the effects of RNA length and RNA sequence we tested the 1–1000nt mutant RNAs (similar or identical lengths only very slightly different sequences and structure with N-protein (Figure 2B).

Thus, we asked which RNA sticker is responsible for conferring the condensation temperature. We observed that only those mutations which resulted in more secondary structure (+dsRNAa–d) lowered the LCST of N-protein with all other mutations having comparable temperature to wildtype (Figure 2B and C). We confirmed the temperature-dependent turbidity results reflected the formation of droplets by examining assemblies under the microscope. Compared to wildtype 1–1000 RNA, +dsRNAa RNA lowered N-protein condensation temperature to 25°C from 37°C (Figure 2D) consistent with the temperature-dependent turbidity results (Figure 2B and C). We confirmed that the dilute phase protein concentration measurement was perfectly anti-correlated with the imaging of the droplets at all temperatures (conditions with larger droplets had lower dilute phase protein concentrations). This suggests higher droplet protein recruitment to droplets for more structure RNA in all conditions consistent with the imaging data (Figure 2E). Thus, long stretches of dsRNA are the RNA sticker which specifies N-protein condensation temperature.

### Phase separation temperature is dependent on RBD2/dsRNA interactions

We next evaluated contributions of RBD1 and/or RBD2 of N-protein in promoting temperature-dependent condensation. We hypothesized that RBD2 would likely be critical based on the ability of dsRNA to tune the LCST behavior. Additionally, N-protein interactions with the 5′UTR increase modestly at higher temperatures in RBD1 mutants (Y109A) as assessed via EMSA (Supplementary Figure S4A and B). This supports that N-protein's RNA binding activity at higher temperatures is independent of RBD1. Furthermore, loss of the putative RBD1 binding site (TRS stem loop), which is described below in detail, had no significant impact on the temperature of condensation (Figure 2B). Similarly, adding an additional RBD1 binding element did not lower the LCST (Supplementary Figure S4C and D) via either a microscopy assay or dilute phase measurement. Together these data support the hypothesis that RBD2/dsRNA encode temperature dependence of N-protein phase separation with RNA.

To test if indeed RBD2 was essential for LCST, we purified an N-protein with a deletion in RBD2 (red amino acids) (Figure 2F) which was predicted to preserve the adjacent conserved dimerization interface (Supplementary Figure S4E). We reasoned that if RBD2-del N-protein had altered phase separation temperature compared to wild-type N-protein than that would confirm a role for RBD2 in specifying temperature. We additionally predicted that wildtype 1–1000 RNA and dsRNA + RNA would have similar condensation temperature with RBD2-Del protein as their interactions with protein would be dictated solely by RBD1 interactions, which are predicted to be equivalent between the two RNA sequences and temperature insensitive. Remarkably, we observed that N-protein RBD2-Del's LCST behavior was significantly altered with both wildtype and +dsRNAa mutant RNA (Figure 2G) compared to wild-type N-protein (Figure 2D) and could phase separate at all tested temperatures even without additional RNA (H_2_O control Figure 2G). Further, similar levels of protein in the dilute phase (*A*_280_ signal) were detected following the phase separation assay for all tested temperatures consistent for altered LCST behavior compared to wildtype (Supplementary Figure S4F). Reducing the N-protein and RNA concentration showed some degree of RNA dependence for the RBD2-Del mutant, but the dramatically lowered LCST behavior was still preserved (Supplementary Figure S4G and H). These data support that RBD2 interactions encode the temperature threshold for phase separation in this system and that in the absence of RBD2 activity, wild type and more structured RNA enhances phase separation similarly.

### RBD2/dsRNA mediated splitting of N-protein dimers promotes condensation

How do N-protein RBD2 dsRNA interactions lower phase separation temperature? We were surprised to see that RBD2 deletion leads to overall enhanced formation of condensates rather than reduction, which is associated with loss of RBD1 activity (26). Based on the literature of SARS-CoV-1 N-protein RBD2 crystal structure (41), we hypothesized that RBD2-del region may stabilize the formation of higher order oligomers of N-protein and the mutant may prevent the fixed stoichiometry dimers and instead promote higher-valence interactions. To address if RBD2-del mutation was destabilizing the formation of N-protein dimers (the reported oligomerization state of N-protein in the absence of nucleic acid (37,54,55)) we performed mass photometry (43). We observed that, consistent with previous studies, wild-type N-protein forms a dimer (Figure 2H) whereas RBD-2 del is mostly a monomer (Figure 2I). We conclude from this that the RBD2-del mutation destabilizes the N-protein dimer which may lead to the reduced temperature and less dependence on RNA for condensation as potentially the monomeric protein is more amenable to multivalent IDR-based interactions. DsRNA addition may mimic the RBD-2 del by destabilizing the dimer of wild-type N-protein at lower temperatures than native sequence (Figure 2J). It is likely that N-protein's RBD2/dimerization domain (and by extension LCST) is under selective pressure as beta sheet 1 and 2 of the dimerization interface (Supplementary Figure S4I and J) are reasonably well conserved in betacoronavirus. Intriguingly, however there are detectable recurrent mutations in patient samples between beta sheets 1 and 2 (Supplementary Figure S4K). It is possible that these mutations or PTMs may alter LCST behavior by destabilizing the dimerization interface.

Collectively, these data suggest that while RBD1 is required for 5′end RNA to phase separate, RBD2 is required for LCST behavior at physiologically-relevant temperature and salt, and RBD2 encodes LCST behavior through preferentially binding to dsRNA. These data reveal exquisite specificity of N-protein for different RNA sequences, raising the question what role this specificity plays in the biology of the virus.

### N-protein RNA interactions that enhance condensation tune translational repression

Given the +dsRNA mutants promoted droplet formation (Figure 1) and the SARS-CoV-2 genome is enriched in dsRNA, even in protein coding sequences (49,56,57), we next asked if N-protein binding and condensation could regulate target RNA translation and if the quality of the dsRNA sticker could tune this regulation. We reasoned the increase in condensation due to dsRNA addition may be antagonistic to translation as some condensates can repress translation (44,58). Thus, an understanding of how N-protein regulates viral RNA translation using its affinity to RNA structure would be informative for the viral life cycle.

**Figure 3.**
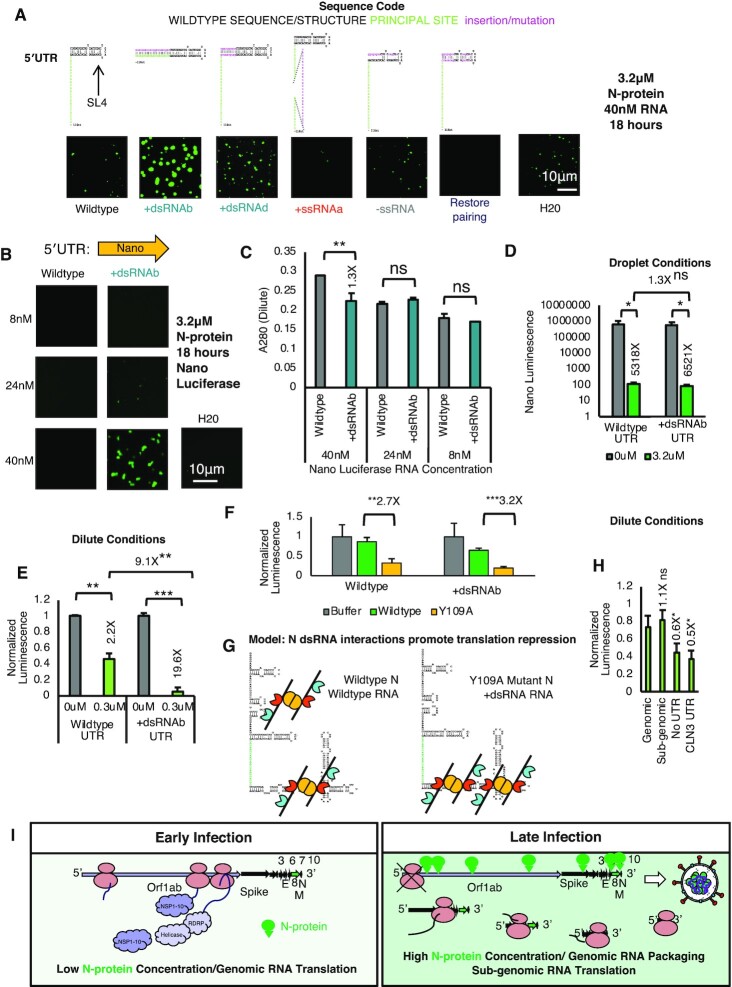
Features which promote N-protein RBD2/dsRNA interactions repress translation. (**A**) Only +dsRNA (teal) mutants enhance condensation in the context of the 5′UTR fragment. All other mutations do not significantly alter condensation. 3.2 uM N-protein (green) 40nM RNA 18 hours of incubation. H2O is water only control. (**B**) Design of luciferase fusion to the 5′UTR of SARS-CoV-2 constructs. Only +dsRNAb UTR: Nano Luciferase undergoes condensation at the highest tested RNA concentration (40 nM/3.2 μM N-protein (green)) (**C**) *A*_280_ absorbance of the remaining protein in the dilute phase from (B). Error bars mark standard deviation for the three replicates and * indicate significance Student's *t* test (**P <0.01, ns not significant) with brackets showing comparison for the indicated statistical test. (**D**) *In vitro* translation assay results for nano luciferase wildtype or more structured fusion constructs. 20-min incubation with 3.2 μM N-protein prior to in vitro translation is sufficient to completely repress translation of nano luciferase. Error bars mark standard deviation for the three replicates and * indicate significance Student's *t* test (** *P*< 0.01, ns not significant) with brackets showing comparison for the indicated statistical test. (**E**) Presence of N-protein condensation promoting RNA structures is associated with reduced translation in dilute phase conditions. Normalized luminescence for nano luciferase constructs (no protein control fluorescent signal is set to 1). Nano luciferase +dsRNAb has a much greater reduction in normalized signal as compared to wildtype. (**F**) Y109A mutant protein which is deficient in RBD1 activity is better able to repress translation than wildtype protein in dilute conditions for both wildtype 5′UTR:Nano and +dsRNAb:Nano. (**G**) Model for N-protein mediated repression of translation via RNA affinity in the dilute phase (limiting protein conditions). Condensation at the structured SL5 inhibits translation and this preferentially occurs in the absence of RBD1 activity or following mutation which enhances RBD2 interactions with SL5 (addition of dsRNA). (**H**) TRS contain sequences (wildtype genomic UTR and subgenomic UTRs of the nucleocapsid gene are less repressed than sequences which do not contain a TRS such as Nano luciferase without a UTR and the 5′ UTR CLN3 from *Ashbya gossypii*. (**I**) Model for N-protein mediated repression of translation via RNA affinity in infection. High affinity sites in the 5′UTR are preferentially occupied by N-protein in early infection to shut down orf1ab translation and switch to packaging. Late-stage infection translation occurs preferentially in sub-genomic RNA.

To address if N-protein mediated protein translational regulation could be encoded by dsRNA, we first needed to design a translation reporter with differential dsRNA content but identical coding sequences (to control for translation). We reasoned that reporter RNAs with different untranslated regions, but identical coding sequences would fulfill our requirements. Thus, we sought to replicate our dsRNA/ssRNA addition experiments (Figure 1) in the context of the SARS-CoV-2 5′UTR (nucleotides 1–267) (Figure 3A) by altering stem-loop 4 (SL4). We observed that only the +dsRNAb (which results in 22nt additional sequence and 44nt of additional dsRNA) drove significant additional condensation relative to wildtype (Figure 3A). +dsRNAd which adds 10 paired nucleotides to the base of SL4 (20 additional nucleotides total) also resulted in minor enhancement. All other non +dsRNA mutants had negligible effects. Thus, length dependent addition of dsRNA to the 5′UTR should be sufficient to enhance condensation independent of the coding sequence when appended in *cis*.

To ask if 5′UTR or +dsRNAb UTR could differentially regulate translation in droplets we fused either the wildtype 5′UTR or a more structured mutant (+dsRNAb) to nano luciferase. To determine if 5′UTR structure affects condensation for the fusions, we mixed 3.2 μM N-protein with 40, 24 or 8 nM RNA. At the highest tested RNA concentration, 40 nM, only the more structured mutant UTR resulted in condensation (Figure 3B). Similarly, only 40 nM RNA condition had a statistically significant difference in *A*_280_ absorbance in the dilute phase suggestive of more protein recruitment to droplets (Figure 3C). Thus, consistent with results above, addition of dsRNA facilitates condensation of nano luciferase fusion RNA.

We then used this system to first ask how condensed conditions (3.2 μM N-protein 40 nM RNA) impact translation? To this end, we performed an in vitro translation assay ±3.2 μM N-protein. We observed that addition of 3.2 μM N-protein almost completely blocked the translation of both tested RNAs (Figure 3D) Collectively, these results suggest that N-protein droplet conditions block translation. We next asked if translation inhibition depended on condensation or N-protein binding in the dilute phase? To this end, we repeated the in vitro translation assay this time with 0.3 μM of N-protein (Figure 3E over 10-fold less N-protein than in Figure 3D). In these conditions, translation of the wild-type UTR was moderately but significantly repressed by 0.3 μM N-protein addition, and the translation of the +dsRNA UTR mutant was almost completely repressed translation (9.1× further reduction in translation compared to wildtype). This is consistent with phase behavior and N-protein affinity differences for these two RNAs (Figure 3B and C). To confirm that translation repression was specified by RBD2/ dsRNA interactions we monitored the degree of translational repression conferred by Y109A mutation N-protein which destroys RBD1 activity. Compared to Wildtype protein control Y109A mutant N-protein resulted in a further reduction of translation for both Wildtype and +dsRNAb UTRs (Figure 3F). Collectively, these data suggest that droplet promoting conditions completely block translation, and conditions where there are no droplets, but still N-protein/RNA interactions can partially block translation. Blocking requires RBD2 rather than RBD1 activity (Figure 3G). Conversely, in limiting N-protein conditions RBD1/UTR interactions can promote translation, as loss of the RBD1 interactions led to a further reduction in translation. We predict this is by redirecting N away from SL5 which contains the start codon suggesting an important role for RBD1 interactions in promoting translation. An important binding site for RBD1 is likely the TRS motif, see further description below (59). In accordance with this model, TRS containing RNAs such as the genomic and nucleocapsid sub-genomic 5′UTR of SARS-CoV-2 are less efficiently repressed than nano luciferase fused to no UTR or a non-SARS UTR such as that of the CLN3 from *Ashbya gossypii* (Figure 3H). Notably, translation repression can be mediated independent of condensation solely based on protein concentration.

We hypothesize that N-protein binding to the genome (particularly the 5′UTR) may act to halt Orf1ab protein translation and promote packaging in later stages on infection (Figure 3I). In support of this idea, N-protein encoding RNA is low at early stages of infection and gradually increases (via generation of sub-genomic N-protein RNA at late stages of infection) (48). This would lead to increased N-protein through time and thereby promoting a switch from translation of Orf1ab in the genome to packaging in late-stage infection. It is likely that individual sgmRNAs tune translation efficiency over the course of infection by utilizing more or less structured UTRs.

### TRS sequence/structure motif promotes N-protein condensation

The data thus far show that a primary driver of phase separation is dsRNA/RBD2 interactions but there are several lines of evidence that suggest additional interactions are mediated by RBD1. First, Y109A mutant N-protein does not undergo condensation with wildtype 5′end sequence. Second, the Y109A + dsRNA droplets have altered morphology suggesting some interaction between N-protein and RNA has been altered in the absence of RBD1 activity. Thus, we sought to identify what RNA sequence features are favored by RBD1.

Given the transcriptional regulatory sequence (TRS in SL3) is the reported binding site of RBD1 in MHV (59), and the 1–500 fragment which contains the TRS was better able to promote phase separation than the 500–1000nt fragment (Figure 1C), we reasoned the TRS may be the preferred binding site of RBD1. Thus, we sought to characterize the importance of the TRS in N-protein condensation (Figure 2).

**Figure 4.**
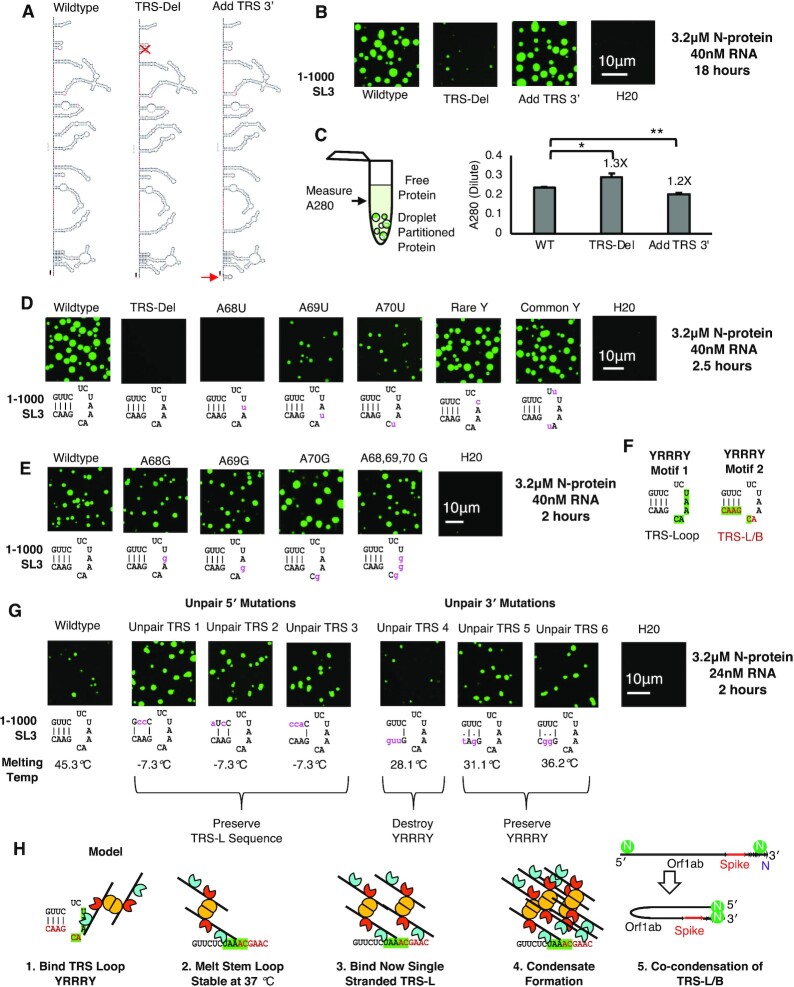
TRS sequence/structure motif promotes N-protein condensation. (**A**) Cartoon of mutations depicted in (B) and (C). TRS-Del deletes SL3 TRS whereas add TRS 3′ adds an additional TRS element to the 3′ end of the RNA. (**B**) 3.2 μM N-protein (green) and 40 nM RNA following 18 hours of incubation for wildtype 1–1000, a mutation which deletes the entire TRS-stem-loop (TRS-del), or a mutation which appends an additional TRS to the 3′ end (Add TRS 3′). (**C**) Add TRS 3′ has lower A280 measurements then wildtype indicative of less protein in solution and more condensation whereas TRS-del is the opposite. Error bars mark standard deviation for the three replicates and * indicate significance Student's *t* test (***P < 0.001, **P < 0.01, ns not significant) with brackets showing comparison for the indicated statistical test. (**D**) 3.2 μM N-protein (green) and 40 nM RNA following 2.5 h of incubation for wildtype 1–1000, a mutation which deletes the entire TRS-stem-loop (TRS-del), A68U mutation, A69U mutation, A70U mutation and mutations which alter the sequence of the A flanking pyrimidines (Y’s) (C’s and U’s) to the most rare and common YYAAAY in the SARS-CoV-2 genome. Deletion of TRS-loop or alteration of the AAA of the loop but not the Y’s leads to a reduction in condensation. (**E**) 3.2 μM N-protein (green) and 40 nM RNA following 2 h of incubation for wildtype 1–1000, A68G mutation, A69G mutation, A70G mutation and mutations which alter the sequence of all three A’s (A68,69,70 G). Do not significantly alter condensation. Suggesting the motif recognized by N-protein is 3 purines flanked by pyrimidine. (**F**) 2 possible YRRRY motifs in SL3, the first is the Loop UAAAC and the second is contained in the TRS-L/B sequence ACGAAC. (**G**) 3.2 μM N-protein (green) and 24 nM RNA following 2 h of incubation for wildtype 1–1000, or mutations which unpair SL3 from the 5′ or 3′ sides. Unpairing SL3 generally enhances condensation (unpair TRS 1, 2, 3, 5 and 6) unless the YRRRY motif is destroyed (Unpair TRS 4). Melting temperature of mutant and wildtype stem loops was calculated using DINAMelt. (**H**) Model for N-protein-SL3 interactions which led to condensation. N-protein RBD1 recognizes the stem loop sequence of SL3, unwinding the stem loop which is stable at 37°C. The now single stranded SL3 is permissive for interaction with the second motif contained in the TRS-L sequence. Location of the two motifs in proximity facilitates condensate formation. Condensate formation at TRS-L/B sequences may promote the genome circularization interaction which is required for sub-genomic RNA generation.

To test if the presence of the TRS was required for phase separation, we deleted the entire TRS stem-loop (TRS-del) or added an additional TRS motifs to the 3′ end (Add TRS-3′) in the context of the 1–1000nt RNA sequence (Figure 4A). TRS-del almost completely blocked phase separation (Figure 4B) and reduced N-protein recruitment to what droplets did form (evident by more N-protein in the dilute phase) (Figure 4C). Conversely, the addition of a TRS-loop (Add TRS-3′) resulted in slightly larger droplets than wildtype and enhanced N-protein recruitment to droplets (Figure 4B). We conclude from these studies that the presence of TRS-loop facilitates N-protein condensation with 5′end RNA.

We next wondered if N-protein could also bind sequences which were similar to the TRS-loop. This is because N-protein can drive condensation with other genomic RNA sequences (26,29,32,33). The TRS-loop sequence, CUAAAC, occurs 16 times across the genome (Supplementary Figure S5C), and a chemically similar sequence YYAAAY (Y = C or U), a TRS-loop-like sequence, occurs 114 times across the genome (Supplementary Figure S5D and E). We hypothesized that the most favored RBD1 binding site was YYAAAY (Y = C or U) which is similar to the TRS-loop sequence and the MHV TRS-L/B sequence CTAAAC. Binding to this sequence is suggested to occur in MHV N-protein experiments (59), molecular dynamics simulations, and experiments with dsDNA (60,61). In accordance with this hypothesis, crosslinking of N-protein is reduced in the region adjacent to the TRS for the RBD1 Y109A mutant N-protein (Supplementary Figure S5A and B (26)). Thus, we tested TRS-del, or mutated the individual As in the TRS-Loop sequence, CUAAAC, (A68U, A69U, A70U named so for the corresponding nucleotides in MHV). We also mutated the Cs and Us in the TRS-Loop sequence based on their occurrence in the SARS-CoV-2 genome. A68U, like TRS-del, completely blocked condensation, and A69U and A70U resulted in a decrease relative to wildtype (Figure 4D). Mutation of sequences to the rare (low frequency in the genome) or common Y (high frequency) sequence had negligible effects on N-protein condensates (Figure 4D) suggesting that either Y is an acceptable nucleotide for binding.

The complete block of condensation by a single point mutant in the A68U RNA was striking and could be due to the primary sequence change or a larger-scale structural rearrangement. To assess a potential structure rearrangement, we performed SHAPE on a subset of the tested RNAs. We observed that compared to wildtype control, there was only a minor change associated with an A68U mutation in the probability to form the wild-type structure in the SL3/TRS (Supplementary Figure S5F) and all stem loops were predicted to form as in the wild-type case. These data indicated that most of the condensate repressive activity was due to primary sequence rather than secondary structure changes.

We next asked if the identity of the A nucleotide is critical for condensation. To test this possibility, we converted A’s in the TRS-Loop sequence to G’s as G is chemically more similar to A then U. We observed that regardless of whether a single A was replaced (A68G, A69G or A70G) or all 3 A’s of the loop were replaced (A68,69,70 G) there was no obvious effect on phase separation (Figure 4E). These data suggest that any sequence of 3 purines flanked by pyrimidines is a potential binding site (YRRRY).

### N-protein binding sites in TRS-B sequences relates to sgmRNA abundance

In examining the genome for YRRRY motifs we noticed that this would include the TRS leader (TRS-L) and TRS body (TRS-B) sequence utilized by SARS-CoV-2 (ACGAAC) (Figure 4F). Therefore, we sought to determine if the TRS-L sequence could drive condensation of N-protein and whether access to this motif was governed by stem loop secondary structure. To this end, we unpaired the TRS-Loop on the 5′ side (preserve ACGAAC) or on the 3′ side (destroy ACGAAC in a way that either destroys or preserves YRRRY). We observed that unpairing the stem loop generally enhanced binding unless the YRRRY motif was destroyed (Figure 4G). Taken together, our data suggest a cooperative binding to the TRS/SL3 governed first by interactions with the Loop sequence which forces the structured SL3 (SHAPE was conducted at 37°C Supplementary Figure S5F) to unpair allowing for additional interaction with the TRS-L. Formation of a small condensate on the now melted TRS duplex could promote long-range RNA–RNA interaction necessary for sgmRNA generation and genome circularization (Figure 4H).

**Figure 5.**
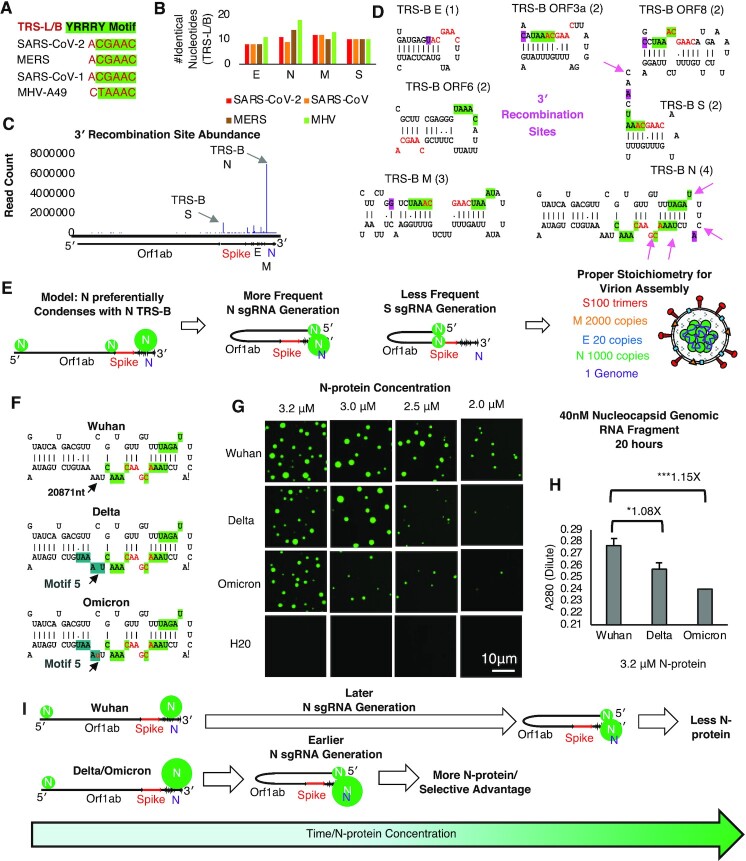
Local YRRRY motif density may control sgmRNA generation ratio. (**A**) Sequence of TRS-L/B for model betacoronaviruses (red text) encompasses the YRRRY motif. (**B**) Similar number of identical nucleotides between TRS-L/B in model coronavirus for structural protein TRS-Bs. (**C**) Variation in abundance of sgmRNA reads in SARS-CoV-2 infected cells. Adapted from (48). (**D**) Sequence and structure of example TRS-B in SARS-CoV-2 genome. Adapted from (49). Red text is the TRS-B sequence ACGAAC. Green highlight is the adjacent YRRRY motifs contained in the stem loop. Of note Orf7ab TRS-B is not included as it is not structured. Magenta highlighted nucleotide is the location of the primary recombination site between TRS-L/and B. Pink arrows refer to less abundant but detectable recombination site. Indicating that nucleocapsid recombination site selection is more degenerate than that of other sgmRNA. Bracketed numbers refer to the number of YRRRY motifs. (**E**) Model of preferential sgmRNA generation dictated by local YRRRY motif abundance. N TRS-B contains more YRRRY motifs (4) than S TRS-B resulting in a higher propensity to form an N condensate at an N gene rather than S and preferential generation of N sgmRNA rather than S. This sgmRNA ratio may allow for proper protein abundance in the assembled virion where there is more absolute number of Nucleocapsid protein molecules than Spike. (**F**) Two independent mutations in Delta (UAAAAU → UAAAU) and Omicron (UAAAAU → UuAAAU) which create a fifth binding site in the in the TRS-B of N. Start codon of Nucleocapsid and overall structure of the TRS-B containing hairpin is not predicted to be altered. (**G**) Fragments of Nucleocapsid RNA containing the TRS-B of N have altered phase behavior following Delta or Omicron mutations in position 20871nt. Morphology is not significantly altered but sequence that contain a fifth binding site exit the phase diagram earlier indicative of higher affinity for RNA. (**H**) A280 absorbance in the dilute phase of the 3.2uM condition shown in figure G shows that sequences which contain five binding sites recruit more protein commensurate with altered phase behavior. Omicron recruits more protein to droplets than Delta and exits the phase diagram sooner. (**I**) Model for how Delta and Omicron mutations in position 20871 may provide a selective advantage for the virus by preferentially generating N sgmRNA earlier and more often at the expense of other sgmRNAs.

We were intrigued by the observation that 2 motifs were in such close proximity in the TRS-L (Figure 4F). Thus, we wondered if a similar YRRRY motif density occurred elsewhere in the genome. To this end we examined TRS-B sequences. TRS-B or body is a conserved (Figure 5A) primary sequence motif found in front of the each of the structural genes in the 3′ side of the genome (spike (S), membrane (M), envelope (E), nucleocapsid (N) etc.). Recombination between the TRS-L and each of the TRS-B is responsible for generation of the sub-genomic RNA required for structural protein production (62). Each TRS-B yields a different structural protein producing RNA. It is unclear how the relative ratios of sgmRNA’s is governed but it is thought to involve base pairing between the TRS-L and the anti-sense TRS-B sequence. In an examination of the number of identical nucleotides between the TRS-L and B in different model betacoronaviruses revealed that the number of identical nucleotides, although substantial, was not very variable between different structural genes (Figure 5B) and could not explain the extreme variation between sgmRNAs in abundance in late-stage infection (Figure 5C) (48).

We wondered if local enrichment of N-protein condensation promoting RNA features might instead explain how TRS-B’s are chosen for sgmRNA generation. To this end, we examined the highly structured (49) TRS-B for YRRRY abundance reasoning that differential N condensation governed by local motif density may promote TRS-B selection. We observed that there was indeed variation in the number of YRRRY motifs in proximity to the TRS-B sequences with TRS-B-E having sequence only in its stem loop and TRS-B-N having four motifs (Figure 5D). We also checked whether YRRRY was more abundant in proximity to the TRS-B than by random chance by performing a one-sided Mann–Whitney *U* test. To this end, we divided the genome into 46 nucleotide bins, removing any bins that overlapped with a TRS-B motif or 20 bp flanking the TRS-B. We observed that the TRS-B containing bins were significantly enriched (*P* < 1.3e–5) in YRRRY motifs comparing to the rest of the genome and similar enrichment was found in the SARS-CoV-1, MERS, and MHV genomes. For SARS-CoV-2, enrichment was preserved even when the TRS-B sequence was not included in the calculation (*P* < 0.014).

We also noticed that the YRRRY motifs tended to be 5′ to the TRS-B sequence. When limiting the enrichment calculation to just the 20 nucleotides proceeding the TRS-B or 26 nucleotide long genome bins the enrichment improved with *P* < 2.5e–7 for TRS-B inclusive and *P* < 0.0011 for YRRRY without TRS-B sequence. Collectively, these results suggest that TRS-B contains densely packed high-quality N-protein binding sites which have both YRRRY (RBD1 motif) in secondary structure (RBD2 motif) compared to the rest of the genome.

We hypothesized that the number of N-protein binding motifs controls the relative abundance of sgmRNAs. Given Nucleocapsid (N) sgmRNA is more abundant than Spike (S) sgmRNA and TRS-B N contains more motifs than TRS-B S, we reasoned that N-protein may preferentially condense with TRS-N and this bias could control the ratio of sgmRNAs present thereby ensuring the proper stoichiometry for virion assembly (Figure 5E). In this model, a single N-protein or a small assembly of N could form just 5′ to the TRS-B tethering it to the TRS-L until the replication transcription complex reaches the junction from the 3′ side. The recombination site could be variable depending on which YRRRY motif the assembly forms. In support of this hypothesis, the TRS-B with the most adjacent YRRRY motifs (N with 4) has the most variable recombination site selection with 4 additional locations (pink arrows) with >5000 reads in infected cells (Figure 5D). Spike in contrast only has one additional site apart from the most abundant (pink highlighted area). This suggests, that to generate more N sub-genomic RNA, SARS-CoV-2 concentrates YRRRY motifs in close proximity to the TRS-B with the consequence of having more variable recombination. This might allow the early and preferential formation of N sgmRNA under limiting N-protein conditions early in infection.

### N-protein YRRRY motifs acquired in Delta and Omicron variants of concern

Operating under the assumption that preferential generation of N sub-genomic RNA may provide a selective advantage to the virus, we next asked if mutations present in highly infectious variants of concern, such as Delta or Omicron, may create additional YRRRY motifs near any TRS-B (particularly that of N). We observed that in position 20871nt, Delta and Omicron independently acquired 2 different mutations that create a fifth YRRRY motif in the TRS-B of N. Delta creates a deletion converting UAAAAU to UAAAU whereas Omicron has an A to U mutation converting UAAAAU to UUAAAU (Figure 5F). Neither of these mutations are predicted to disturb secondary structure (being in an unpaired region of the stem loop) or alter the start codon of the N gene. We reasoned that the additional YRRRY motif in Delta and Omicron's TRS-B may alter the condensation of N-protein. To this end, we synthesized an RNA fragment containing the TRS-B of N protein for both Wuhan as well as a single nucleotide difference associated with Delta and Omicron but otherwise the RNAs are identical. We observed that at high protein to RNA ratios there was no obvious difference in the morphology or size of the droplets (Figure 5G) although Delta and Omicron did have less protein in the dilute phase, indicating more recruitment of N to the dense phase (Figure 5H). Lowering the protein to RNA ratio revealed that Delta and Omicron RNAs phase separated in different concentrations than Wuhan. Specifically, Delta and Omicron both showed re-entrant phase behavior (63) where N-protein becomes soluble as opposed to condensed at lower RNA concentrations. This phase behavior is indicative of higher affinity of the RNA for N-protein from the Delta and Omicron variants.

Collectively, these results suggest that preferential co-condensation between N-protein and the TRS-B of N early in infection under limiting N-protein conditions, may provide a selective advantage to SARS-CoV-2 allowing for earlier generation of N-protein to support multiple N-protein mediated functions required for viral replication. Mutations which create additional N-protein condensation promoting YRRRY motifs may lower the protein threshold required for condensation driving the recombination event to occur earlier in infection (Figure 5I).

### RNA sequence/structure may encode N-protein genome interactions to pattern RNP formation in virions

**Figure 6.**
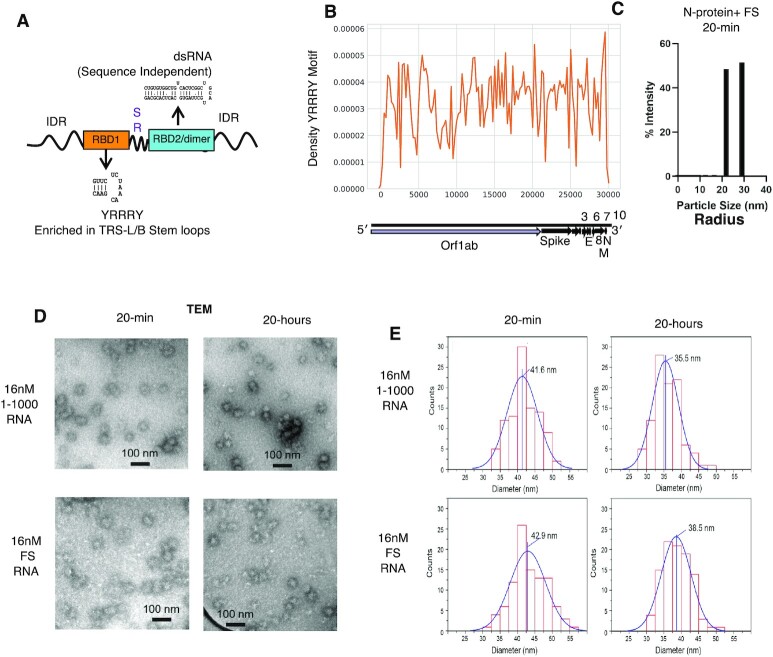
RNA sequence/ structure encodes N-protein genome interactions. (**A**) Model of RNA sequence preferences of SARS-CoV-2 N-protein RNA binding domains 1 (orange) and 2 (blue). RBD1 (teal box) binds TRS-like (YRRRY) sequences in a structure dependent manner. RBD2/dimerization domain (blue box) binds dsRNA in a sequence independent manner. (**B**) Density of YRRRY motif (orange) across the SARS-CoV-2 genome. (**C**) Dynamic light scattering of 16 nM FS RNA and 4 μM protein. Following 20-minutes of incubation results in particles of ∼21.9 or 29 nm radius (∼43.7–58 nm in diameter). (**D**) Representative TEM images of small clusters which form from a mixture of 4μM N-protein and either 16nM FS or 16 nM 1–1000 5′end when incubated for 20 min or 20 h at room temperature. Scale bar is 100 nM. (**E**) Quantification of small clusters as depicted in panel D. for 1–1000 5′end, or FS. Clusters shrink by ∼15% following 20 h of incubation.

Given the key central role of N-protein in genome packaging, we next asked how what we have learned thus far about different types of N-protein/RNA interactions may impact packaging. Particularly, we were interested in the patterning of the YRRRY motif of RBD1 (Figure 6A) given our observation that there were 813 of these present in the genome. If there are 1000 Nucleocapsid's per virion (64), this could indicate that this motif is heavily utilized in virion assembly. As expected, we observed local abundance of YRRRY motifs surrounding notable TRS-B motifs but a uniform abundance of motifs across the genome (Figure 6B).

Based on high-resolution cryo-EM tomography, the genome of SARS CoV-2 is arranged inside virions in a so-called ‘birds-nest’ arrangement with ‘eggs’ made of RNP complexes that are ∼14–20 nm (65,66). We previously observed that RNA derived from the center of the SARS-CoV-2 genome including RNA encoding the Frameshifting-region (FS) promoted N-protein solubilization at the microscopic level (26). We reasoned that the solubilizing effect of FS RNA may be conferred by the formation of diffraction limited clusters that may be distinct from condensation or are arrested from coarsening into macroscopic droplets. If indeed small RNP-scale particles form in this cell free system this would indicate that N-protein binding to RNA, as dictated by RNA sequence, was sufficient to condense RNA independent of cellular machinery.

To address if N-protein mediated condensation is sufficient to compact RNA to RNP- size assemblies cell free, we first asked what size particles form from FS RNA (1000nt in length)? We examined FS RNA as this RNA does not drive macroscopic condensation at 4 μM N-protein and 16–24 nM RNA at room temperature (Figure 2A). To this end, we measured the particles formed from 16 nM FS RNA and 4 μM N-protein by dynamic light scattering. We chose 250:1 protein to RNA as this would be reminiscent of late-stage infection and packaging (48). We observed that following 20-minute incubation time at room temperature FS RNA forms homogenously sized clusters 44–58 nM in diameter (Figure 6C) suggesting RNA cluster generation can occur cell free and in conditions which do not support RBD2/dsRNA interactions.

To directly visualize cluster formation a second way, we used TEM. Indeed, after 20-min of incubation a relatively monodispersed population of symmetric, circular assemblies form that are centered on 42.9 nm diameter (Figure 6D). To assess if these formations were specific to FS RNA, we also examined N-protein 1–1000 RNA in conditions that do not support phase separation (room temperature). These formed similarly shaped and sized particles as the FS RNA (Figure 6D). The assemblies formed with both RNAs are more than double the size of the reported RNP (∼14–20 nM) diameter (65,66).

We wondered what caused the >2-fold size discrepancy between these RNP assemblies and the RNPs seen in virions? It is established that some droplets age into gel-like or glass-like states that can be associated with compaction (67), we therefore asked how the clusters change with time. Indeed, at the 20-h time point smaller, more similar sized clusters for both RNAs were formed (Figure 6D) indicating the clusters are shrinking by ∼15% over time, independent of RNA sequence (Figure 6E). Some larger, rarer clusters were detected at 20 h for both RNAs (Supplementary Figure S6A and B). Thus, N-protein and 1 kb gRNA form monodispersed clusters cell free that compact over time. Both RNA and protein are required to form clusters (Supplementary Figure S6C). The similar size distribution of 5′end and FS fragments may result from the similar length (1 kb) and overall affinity for RBD1 (the temperature insensitive RNA binding domain). Therefore, condensation differences between 5′end and FS require temperature-sensitive RBD2 interactions.

We postulated that FS interactions with N-protein may be heavily dependent on RBD1 rather than RBD2. In support of this hypothesis, the FS RNA contains 24 YRRRY and FS does not engage with RBD2 in a way that alters condensation temperature (Figure 2A). To confirm FS N-protein interactions are strongly RBD1 dependent, we performed RNP-map on FS with wildtype and Y109A mutant (RBD1 deficient) N-protein (Supplementary Figure S6D and E). We observed that the majority of the N-protein crosslinking peaks in FS were absent following incubation with Y109A mutation. Some Y109A-independent crosslinking was detected (purple boxes) and this tended to be adjacent to structured RNA. Thus, FS/N-protein interactions are primarily driven by RBD1 (Supplementary Figure S6D and E). RBD1 binding site patterning conferred by structured YRRRY motifs may be required for RNP-sized cluster generation.

## DISCUSSION

**Figure 7.**
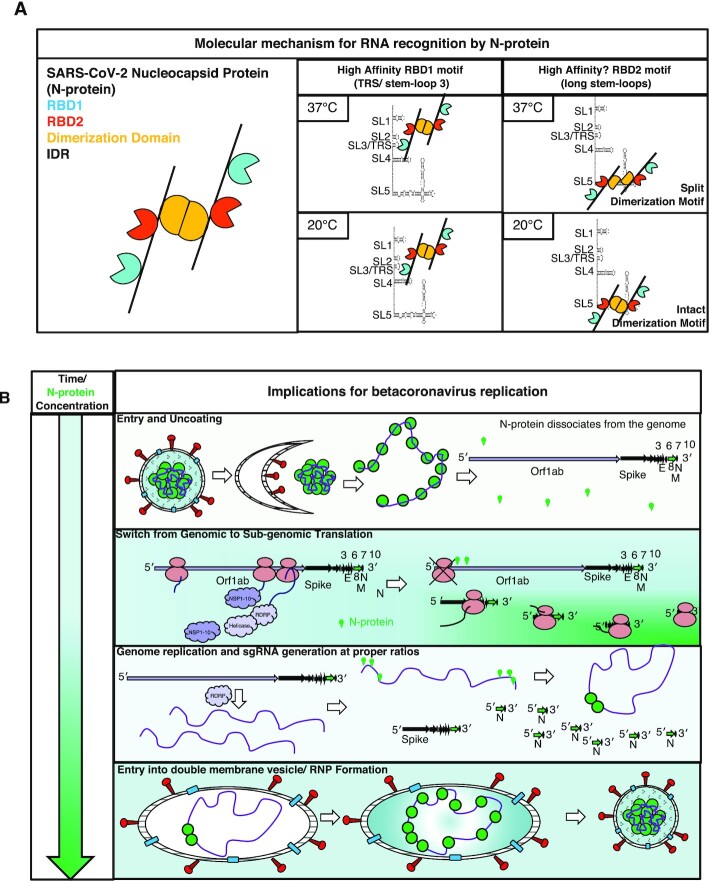
Model: Molecular mechanism and implications for betacoronavirus replication. (**A**) N-protein's two RNA binding domains prefer two dsRNA dependent RNA stickers. RBD1 (teal) binds TRS stem–loop (and similar sequences) with high affinity. RBD2 (dark orange) binds long stem–loops in a temperature dependent manner. (**B**) Time dependent accumulation of N-protein specifies N-protein's multiple roles in betacoronavirus by tuned patterned affinity for the two dsRNA dependent RNA stickers. High affinity sites (genome ends) are occupied preferentially early in infection when N-protein concentrations are low. Low affinity sites (genome center) are occupied late in infection when N-protein concentrations are high. Occupation of high affinity sites at genome ends promotes the switch from genome translation to circularization and ultimately packaging.

In this paper, we elucidate the RNA sequence and structure preferences of SARS-CoV-2 N-protein condensation to understand how these features lead to condensate properties relevant to viral processes in cells. We show that (i) RBD2 prefers dsRNA in a sequence-independent manner (ii) RBD1 prefers TRS-like sequences in an RNA structure-dependent manner (Figure 7A). We elucidate emergent properties conferred by the two ‘RNA stickers’ for SARS-CoV-2 N-protein to understand how these features lead to distinct condensate properties that could control different viral processes in cells. We show that RNA sequence/structure features specify N-protein interactions to regulate LCST behavior that can impact translation, sgmRNA generation and gRNA RNP cluster size using in a cell-free model This is suggestive of a model by which cells exploit RNA sticker patterning and quality to perform multiple, distinct N-protein dependent functions (Figure 7B).

### RBD2 prefers dsRNA in a sequence independent manner

Addition of dsRNA, independent of sequence (Figure 1D–F, Supplementary Figure S1A), resulted in more condensation in all tested conditions (Supplementary Figure S1C–E). Reduction or addition of short ssRNA (comparable lengths to dsRNA mutants) sequences resulted in negligible enhancement of the number/size of droplets (Figure 1D–F, Supplementary Figure S1B). Unpairing dsRNA generally reduced formation of condensates (Figure 1D–F). There is likely an absolute length preference for RBD2 binding (Supplementary Figure S1A) which is consistent with 5′UTR stem-loop length altering experiments leading to viral plaque reduction (68,69). The lack of observed primary sequence specificity to N-protein RBD2 dsRNA binding (Figure 1D–F) may explain why previous stem-loop swap experiments, switching stem-loops from one betacoronavirus for another, generated functional virus (70–72). Although dsRNA length is important for RBD2 engagement, the specific sequence of the stem-loops is not critical (Figure 1D–F) suggesting that N-protein may be able to engage with the entirety of the highly structured genome of SARS-CoV-2 (49,56,57). The lack of dsRNA sequence specificity is also suggested by the nature of the RBD2 motif, a lysine-rich IDR, which is unlikely to have primary RNA sequence specificity. Therefore, these data suggest that the sequence of the stem–loop does not matter for viral production and but only minor differences in length are tolerated.

### RBD2 dsRNA interactions encode N-protein LCST behavior

We postulate that the native, more structured stem-loops of the genome ends (i.e. SL5, 13 and 14 in the 5′end) are the most efficient binding sites for RBD2 (as evidenced by RBD1 independent crosslinking adjacent to these stem-loops (26)) and this binding promotes condensation at human body temperature (37°C) by facilitating N-protein dimer splitting. We predict that the binding to RBD2 in combination with physiological temperature (37°C) allows for the dissolution of the dimerization domain adjacent to RBD2 (Figure 2J). Temperature is likely to facilitate the ‘unfolding’ of the dimerization domain as purified RBD2-dimerization domain undergoes a structural change at ∼50°C by differential scanning fluorometry (51). Of note, the temperature of dimerization unfolding determined by Zinzula et al. for purified RBD2 dimerization domain alone is very close to our observed full-length N-protein without RNA turbidity temperature (46°C Figure 2A) suggesting temperature-dependent unfolding of this domain is critical to LCST behavior. The exposure of the hydrophobic core of the dimerization domain to the solution following temperature and RNA engagement may facilitate condensation as hydrophobic regions tend to be insoluble.

The temperature at which dimerization occurs can be lowered via addition of dsRNA, potentially due to increasing the overall affinity of wildtype N-protein's two RBD2 domains or by offering additional sites of interaction (at a greater distance apart) on the same stem-loop. In support of the latter possibility, the two RNA binding domains are arranged diagonal to each other, and dsRNA binding may force the dimer apart (Figure 2J). Cryo-EM data of purified RBD-2/dimerization domain with ssRNA seems to agree with this hypothesis (51) with 7 base pairs of ssRNA spanning between two RBD2 motifs and a marked separation in the dimer region. Chemical shift displacement experiments yielded similar results, in which longer DNA oligos further alter the resonance of the amino acids in beta sheet 2 of the dimerization interface (73).

Our results also may explain why not all labs reporting N-protein condensation have observed LCST behavior in N-protein RNA interactions as these results show that LCST behavior is specifically encoded in N-protein dsRNA interaction. N-protein dsRNA interaction is unlikely to be observed in reconstitution experiments conducted with less physiological, unstructured RNA (poly U for example). RBD2 also seems to regulate the dimerization domain of N-protein (55). RBD2 dimerization is highly dependent on the salt concentration with only physiological salt concentrations (150 ± ∼30 mM) allowing for LCST behavior (26,33,55). Lower salt results in an increase in N-protein dimerization domain adjacent interactions (32) which might increase the required total solution concentration of N-protein for phase separation, thus also increasing the temperature boundary of the LCST behavior. Others have not observed LCST behavior using nearly identical RNA sequences and N-protein preparation methods, but they were using much lower salt (33,74). We conclude that because physiological levels of salt are more likely to be present in cells, LCST behavior of N-protein is relevant.

As RBD2 may recognize RNA through a complex interaction involving charge, disorder, and transient protein structure. It is highly likely that post-translational modifications (PTMs) play a significant role not only in RBD-2 binding RNA but also dimerization and LCST behavior. This may begin to explain why those labs which purify N-protein from mammalian cells did not observe LCST while those which purify N-protein from bacterial sources did (26,32,55). As packaged N-protein is specifically free of post-translational modification (75,76), we hold that the LCST behavior is likely still relevant for packaging with other N-protein compartments such as those regulating viral RNA transcription and translation being far more likely candidates to be regulated by PTMs (particularly those droplets that form outside double membrane vesicles associated with packaging). Future directions will explore how PTMs tune condensation temperature to potentially sustain viral replication and viral RNA N-protein interactions during late-stage infection/fever temperatures. Of particular interest, is the conserved Tyrosine of beta sheet 2 of the dimerization interface (Supplementary Figure S4I and J).

Additionally, our results suggest the primary sequence of RBD2 dimerization region is critical for RNA binding and LCST behavior we would postulate that any mutation that arises and is selected for in these regions (in patient isolates or across species) would be particularly informative. Most patient samples appear in the SR rich region of N-protein with comparatively few mutations present in structured regions and RNA binding domains (77). P326 and S327 of N-protein located between beta sheets 1 and 2 of the dimerization interface are recurrently mutated in patients (Supplementary Figure S4K). S327 can also be phosphorylated (78). We would postulate that these mutations may have altered LCST behavior given their location.

### RNA stem loop length is under selective pressure to allow for condensation at 37°C

Importantly, our results suggest excessive differences in the length of the stem-loops appears to alter temperature encoding behavior with ∼20–24nt of dsRNA (present in SL5, SL12 and SL13 encoding condensation at 37°C and additional dsRNA (10nt+ - 80nt+) lowering the temperature to as low as 25°C (Figure 2B and C). The absolute length of the stem-loop must be under a degree of selective pressure. Importantly, the most structured stem-loops (5, 12 and 13) are highly conserved (74)) suggesting that stem–loop length mediated regulation is a universal feature for proper viral production, but subtle differences exist in the stem–loop length between individual viruses. This matches with experiments in MHV virus where altering the pairing of stem–loop 1 reduced the efficiency of viral production (79). This suggests that stem-loop length may be co-evolving with N-protein RBD2/dimerization sequence, protein amount or both.

### RBD1 prefers TRS-like sequences in an RNA structure-dependent manner

RBD1 preferentially crosslinked adjacent to SL3/TRS in the first 1000nt of the SARS-CoV-2 genome (26) (Supplementary Figure S5A and B). Our model suggests that two primary sequence motifs (YRRRY) contained within SL3, the loop and the TRS-L enhance N-protein condensation. All TRS-B motifs (save Orf7A and B) (Figure 5D) are similarly double stranded in the genome and contain varying numbers YRRRY motifs with E containing a single motif and N containing 4 in the original Wuhan isolate of SARS-CoV-2. The variable enrichment of RBD1 condensate promoting YRRRY motifs is correlated to the relative abundance of sgmRNAs in the transcriptome of SARS-CoV-2 infected cells (Figure 5C) with Nucleocapsids sgmRNA being the most abundant with the most condensation promoting motifs. This suggests a model where preferential condensation at the TRS-B of N may promote N sgmRNA generation over other sgmRNAs. Mutations in variants of concern create additional condensate promoting motifs in the TRS-B of N which alter the phase behavior and may allow for the earlier and more frequent generation of N sgmRNA to produce N-protein. Mutations which enhance N-protein total amount (80) or addition of accessory N-protein (59) enhance viral replication by plaque assay. Collectively, these results suggest that preferential production of N sgmRNA’s provides a selective advantage to SARS-CoV-2.

### RNA sequence/structure features encode N-protein interactions to regulate RNA condensation

Distinct N-protein ‘stickers’ are distributed throughout the genome (Figure 6B). This prompted us to hypothesize N-protein sticker patterning could be relevant for packaging. Although N-protein clearly has tendencies to form macroscopic condensates in vitro and in cells, the packaged genome is instead packed into regularly-spaced RNPs which may be arrested in coarsening. Reconstituted N-protein mixed with 1000nt RNA fragments in physiological salt and pH was able to form clusters that were roughly 1.75X-2.5X the diameter of the RNP, the unit of packaging of the virion (Figure 6D and E). This size difference suggests that either (i) the RNA content of the RNP is ∼500–1000nt (to give a 14–20 nm RNP diameter) or (ii) further, compaction occurs in the cells. We suggest the former possibility is more likely as there is a number range of RNPs (30–35 by cryo-EM suggesting each RNP must contain less then 1000nt (∼30 kb genome/∼35 RNPs) and there is likely a flexible linker region composed of RNA depleted in N-protein (less electron dense) between each RNP to facilitate compaction. Additionally, ∼500–1000 is the approximate size of the majority of the topological organization within the SARS-CoV-2 virion (81). These data suggest that the information needed to condense the RNA genome is contained within the genomic RNA sequence.

Future directions will involve modeling of the SARS-CoV-2 dsRNA and structured TRS-loop-like sequences patterning across the genome to examine if indeed sequence element patterning is sufficient for RNP patterning (12,82). Of note, the length of viral RNA fragments tested in this work (0.5 and 1 kb) is highly relevant for this consideration as each RNP/egg is likely to contain <1 kb. We and others have observed that longer RNAs (32) including RNA purified from infected cells containing SARS-CoV-2 genome (26) results in a ‘string of pearls’ type droplets rather than rounded droplets further suggesting that the formation of RNPs/eggs is recapitulated cell free. Thus, the fragments tested here are short enough to encode single RNP/egg like features but long enough to have sequence and structural complexity to allow for observable regional differences in condensation.

### N-protein accumulation regulates infection by tuned/patterned affinity for dsRNA stickers

Our data (Figure 7) suggests a mechanism by which N-protein can perform multiple distinct functions over time in the same cytoplasm depending on N-protein concentration. Following viral entry, N-protein concentration is low. The low protein concentration allows for N-protein to dissociate from the condensed genome and for the initiation of translation. As infection progresses, N-protein's (and other structural proteins) accumulation is driven by production from sub-genomic transcripts (48,83). The accumulation of N-protein initiates a switch from translation to packaging, shutting down non-structural protein production while sparing the sub-genomic RNAs (which lack the most structured stem-loops 5 and on) (48,56). The structure of the RBD1 motif, the TRS, on the sub-genomic RNA is also predicted to regulate translation in a structure dependent manner with unstructured TRS present on the highly translated N-protein sub-genomic RNA (56,83).The enrichment of high affinity N-protein binding sites at genome ends may allow for condensation-mediated circularization to promote single genome packaging (26,82). Finally, within double membrane vesicles, RNPs form as additional N-protein accumulates over time, with high concentration driving N-protein recruitment to low affinity sites in the genome center. The condensed genome ultimately matures into virions. We have identified unique dsRNA encoded ‘stickers’ for N-protein conferred by the two RNA binding domains. The patterning and quality of the two N-protein dsRNA stickers can confer N-protein's multiple functions through concentration dependent binding and condensation. Thus, biochemical complexity needed for viral replication can be achieved with minimal components.

### Considerations for other RNA-based phase separation

Notably, increasing RNA order, rather than disorder, through additional RNA structure drives N-protein condensation. These results contrast with those observed by the Mayr lab where increased disordered, single-stranded regions in RNA promoted intramolecular association and condensation (84). This discrepancy is likely due to differences in the proteins, specifically the preference of both of N-protein's RNA binding domains for the highly-structured RNA genome of SARS-CoV-2 (dsRNA stickers).

Our work suggests that reconstitution experiments of phase separating proteins with similar dsRNA preferences must be carried out with physiological RNA targets to capture biological behavior. DsRNA–protein interactions are not captured with poly A or U. In short, RNA sequence and structure profoundly influence the behavior of phase-separating systems. Finally, this work shows the complexity of the RNA–protein code in determining the kinetics, and emergent properties of biomolecular condensates. We predict that this is the tip of the iceberg in terms of unraveling the information provided by RNA sequence to specify the form and function of condensates.

## DATA AVAILABILITY

All data are available upon request from C.A.R. or A.S.G. Raw and analyzed RNP-map and SHAPE data are available here GEO: GSE162569 and https://www.ncbi.nlm.nih.gov/sra/?term = PRJNA830870.

## Supplementary Material

gkac596_Supplemental_Files

## References

[1] Boeynaems S. , Alberti S., Fawzi N.L., Mittag T., Polymenidou M., Rousseau F., Schymkowitz J., Shorter J., Wolozin B., Van Den Bosch L. et al. Protein phase separation: a new phase in cell biology. Trends Cell Biol. 2018; 28:420–435. 29602697 10.1016/j.tcb.2018.02.004 PMC6034118

[2] Hyman A.A. , Weber C.A., Jülicher F. Liquid-liquid phase separation in biology. Annu. Rev. Cell Dev. Biol. 2014; 30:39–58. 25288112 10.1146/annurev-cellbio-100913-013325

[3] Alberti S. , Gladfelter A., Mittag T. Considerations and challenges in studying liquid-liquid phase separation and biomolecular condensates. Cell. 2019; 176:419–434. 30682370 10.1016/j.cell.2018.12.035 PMC6445271

[4] Kar M. , Dar F., Welsh T.J., Vogel L., Kühnemuth R., Majumdar A., Krainer G., Franzmann T.M., Alberti S., Seidel C.A.M. et al. Phase separating RNA binding proteins form heterogeneous distributions of clusters in subsaturated solutions. Proc. Natl. Acad. Sci. U.S.A. 2022; 119:e2202222119. 35787038 10.1073/pnas.2202222119 PMC9282234

[5] Mittag T. , Pappu R.V. A conceptual framework for understanding phase separation and addressing open questions and challenges. Mol. Cell. 2022; 82:2201–2214. 35675815 10.1016/j.molcel.2022.05.018 PMC9233049

[6] Choi J.M. , Holehouse A.S., Pappu R.V. Physical principles underlying the complex biology of intracellular phase transitions. Annu. Rev. Biophys. 2020; 49:107–133. 32004090 10.1146/annurev-biophys-121219-081629 PMC10715172

[7] Rubinstein M. , Semenov A.N. Thermoreversible gelation in solutions of associating polymers. 2. Linear dynamics. Macromolecules. 1998; 31:1386–1397.

[8] Semenov A.N. , Rubinstein M. Thermoreversible gelation in solutions of associative polymers. 1. Statics. Macromolecules. 1998; 31:1373–1385.

[9] Wang J. , Shi C., Xu Q., Yin H. SARS-CoV-2 nucleocapsid protein undergoes liquid–liquid phase separation into stress granules through its N-terminal intrinsically disordered region. Cell Discov. 2021; 7:3–7. 33479219 10.1038/s41421-020-00240-3 PMC7817956

[10] Wang J. , Choi J.M., Holehouse A.S., Lee H.O., Zhang X., Jahnel M., Maharana S., Lemaitre R., Pozniakovsky A., Drechsel D. et al. A molecular grammar governing the driving forces for phase separation of Prion-like RNA binding proteins. Cell. 2018; 174:688–699. 29961577 10.1016/j.cell.2018.06.006 PMC6063760

[11] Martin E.W. , Holehouse A.S., Peran I., Farag M., Incicco J.J., Bremer A., Grace C.R., Soranno A., Pappu R.V., Mittag T. Valence and patterning of aromatic residues determine the phase behavior of prion-like domains. Science. 2020; 367:694–699. 32029630 10.1126/science.aaw8653 PMC7297187

[12] Choi J.M. , Dar F., Pappu R.V. LASSI: a lattice model for simulating phase transitions of multivalent proteins. 2019; 10.1371/journal.pcbi.1007028 PMC6822780 31634364

[13] Vernon R.M.C. , Chong P.A., Tsang B., Kim T.H., Bah A., Farber P., Lin H., Forman-Kay J.D. Pi-Pi contacts are an overlooked protein feature relevant to phase separation. Elife. 2018; 7:e31486. 29424691 10.7554/eLife.31486 PMC5847340

[14] Bremer A. , Farag M., Borcherds W.M., Peran I., Martin E.W., Pappu R.V, Mittag T Deciphering how naturally occurring sequence features impact the phase behaviors of disordered prion-like domains. Nat. Chem. 2022; 14:196–207. 34931046 10.1038/s41557-021-00840-w PMC8818026

[15] Nott T.J. , Petsalaki E., Farber P., Jervis D., Fussner E., Plochowietz A., Craggs T.D., Bazett-Jones D.P., Pawson T., Forman-Kay J.D. et al. Phase transition of a disordered nuage protein generates environmentally responsive membraneless organelles. Mol. Cell. 2015; 57:936–947. 25747659 10.1016/j.molcel.2015.01.013 PMC4352761

[16] Roden C. , Gladfelter A.S. RNA contributions to the form and function of biomolecular condensates. Nat. Rev. Mol. Cell Biol. 2021; 22:183–195. 32632317 10.1038/s41580-020-0264-6 PMC7785677

[17] Guseva S. , Milles S., Jensen M.R., Salvi N., Kleman J.P., Maurin D., Ruigrok R.W.H., Blackledge M. Measles virus nucleo- and phosphoproteins form liquid-like phase-separated compartments that promote nucleocapsid assembly. Sci. Adv. 2020; 6:eaaz7095. 32270045 10.1126/sciadv.aaz7095 PMC7112944

[18] Brocca S. , Grandori R., Longhi S., Uversky V. Liquid–liquid phase separation by intrinsically disordered protein regions of viruses: roles in viral life cycle and control of virus–host interactions. Int. J. Mol. Sci. 2020; 21:9045. 10.3390/ijms21239045 PMC7730420 33260713

[19] Heinrich B.S. , Maliga Z., Stein D.A., Hyman A.A., Whelan S.P.J. Phase transitions drive the formation of vesicular stomatitis virus replication compartments. MBio. 2018; 9:10.1128/mBio.02290-17. PMC6123442 30181255

[20] Nikolic J. , Le Bars R., Lama Z., Scrima N., Lagaudrière-Gesbert C., Gaudin Y., Blondel D. Negri bodies are viral factories with properties of liquid organelles. Nat. Commun. 2017; 8:58. 28680096 10.1038/s41467-017-00102-9 PMC5498545

[21] Rincheval V. , Lelek M., Gault E., Bouillier C., Sitterlin D., Blouquit-Laye S., Galloux M., Zimmer C., Eleouet J.F., Rameix-Welti M.A. Functional organization of cytoplasmic inclusion bodies in cells infected by respiratory syncytial virus. Nat. Commun. 2017; 8:563. 28916773 10.1038/s41467-017-00655-9 PMC5601476

[22] Monette A. , Niu M., Chen L., Rao S., Gorelick R.J., Mouland A.J. Pan-retroviral nucleocapsid-mediated phase separation regulates genomic RNA positioning and trafficking. Cell Rep. 2020; 31:107520. 32320662 10.1016/j.celrep.2020.03.084 PMC8965748

[23] Alenquer M. , Vale-Costa S., Etibor T.A., Ferreira F., Sousa A.L., Amorim M.J. Influenza A virus ribonucleoproteins form liquid organelles at endoplasmic reticulum exit sites. Nat. Commun. 2019; 10:1629. 30967547 10.1038/s41467-019-09549-4 PMC6456594

[24] McBride R. , van Zyl M., Fielding B.C. The coronavirus nucleocapsid is a multifunctional protein. Viruses. 2014; 6:2991–3018. 25105276 10.3390/v6082991 PMC4147684

[25] Chang C.K. , Hou M.H., Chang C.F., Hsiao C.D., Huang T.H. The SARS coronavirus nucleocapsid protein - Forms and functions. Antiviral Res. 2014; 103:39–50. 24418573 10.1016/j.antiviral.2013.12.009 PMC7113676

[26] Iserman C. , Roden C.A., Boerneke M.A., Sealfon R.S.G., McLaughlin G.A., Jungreis I., Fritch E.J., Hou Y.J., Ekena J., Weidmann C.A. et al. Genomic RNA elements drive phase separation of the SARS-CoV-2 nucleocapsid. Mol. Cell. 2020; 80:1078–1091. 33290746 10.1016/j.molcel.2020.11.041 PMC7691212

[27] Scherer K.M. , Mascheroni L., Carnell G.W., Wunderlich L.C.S., Makarchuk S., Brockhoff M., Mela I., Fernandez-Villegas A., Barysevich M., Stewart H. et al. SARS-CoV-2 nucleocapsid protein adheres to replication organelles before viral assembly at the Golgi/ERGIC and lysosome-mediated egress. Sci. Adv. 2022; 8:eabl4895. 34995113 10.1126/sciadv.abl4895 PMC10954198

[28] Cascarina S.M. , Ross E.D. A proposed role for the SARS-CoV-2 nucleocapsid protein in the formation and regulation of biomolecular condensates. FASEB J. 2020; 34:9832–9842. 32562316 10.1096/fj.202001351 PMC7323129

[29] Carlson C.R. , Asfaha J.B., Ghent C.M., Howard C.J., Hartooni N., Safari M., Frankel A.D., Morgan D.O. Phosphoregulation of phase separation by the SARS-CoV-2 N protein suggests a biophysical basis for its dual functions. Mol. Cell. 2020; 80:1092–1103. 33248025 10.1016/j.molcel.2020.11.025 PMC7677695

[30] Chen H. , Cui Y., Han X., Hu W., Sun M., Zhang Y., Wang P.H., Song G., Chen W., Lou J. Liquid–liquid phase separation by SARS-CoV-2 nucleocapsid protein and RNA. Cell Res. 2020; 30:1143–1145. 32901111 10.1038/s41422-020-00408-2 PMC7477871

[31] Cubuk J. , Alston J.J., Incicco J.J., Singh S., Stuchell-Brereton M.D., Ward M.D., Zimmerman M.I., Vithani N., Griffith D., Wagoner J.A. et al. The SARS-CoV-2 nucleocapsid protein is dynamic, disordered, and phase separates with RNA. Nat. Commun. 2021; 12:1936. 33782395 10.1038/s41467-021-21953-3 PMC8007728

[32] Jack A. , Ferro L.S., Trnka M.J., Wehri E., Nadgir A., Costa K., Schaletzky J., Yildiz A. SARS cov-2 nucleocapsid protein forms condensates with viral genomic RNA. PLOS Biol. 2021; 19:e3001425. 34634033 10.1371/journal.pbio.3001425 PMC8553124

[33] Lu S. , Ye Q., Singh D., Cao Y., Diedrich J.K., Yates J.R., Villa E., Cleveland D.W., Corbett K.D. The SARS-CoV-2 nucleocapsid phosphoprotein forms mutually exclusive condensates with RNA and the membrane-associated M protein. Nat. Commun. 2021; 12:502. 33479198 10.1038/s41467-020-20768-y PMC7820290

[34] Perdikari T.M. , Murthy A.C., Ryan V.H., Watters S., Naik M.T., Fawzi N.L. SARS-CoV-2 nucleocapsid protein phase-separates with RNA and with human hnRNPs. EMBO J. 2020; 39:e106478. 33200826 10.15252/embj.2020106478 PMC7737613

[35] Savastano A. , Ibáñez de Opakua A., Rankovic M., Zweckstetter M. Nucleocapsid protein of SARS-CoV-2 phase separates into RNA-rich polymerase-containing condensates. Nat. Commun. 2020; 11:6041. 33247108 10.1038/s41467-020-19843-1 PMC7699647

[36] Zhao M. , Yu Y., Sun L.M., Xing J.Q., Li T., Zhu Y., Wang M., Yu Y., Xue W., Xia T. et al. GCG inhibits SARS-CoV-2 replication by disrupting the liquid phase condensation of its nucleocapsid protein. Nat. Commun. 2021; 12:2114. 33837182 10.1038/s41467-021-22297-8 PMC8035206

[37] Forsythe H.M. , Rodriguez Galvan J., Yu Z., Pinckney S., Reardon P., Cooley R.B., Zhu P., Rolland A.D., Prell J.S., Barbar E. Multivalent binding of the partially disordered SARS-CoV-2 nucleocapsid phosphoprotein dimer to RNA. Biophys. J. 2021; 120:2890–2901. 33794152 10.1016/j.bpj.2021.03.023 PMC8007181

[38] Zhang H. , Elbaum-Garfinkle S., Langdon E.M., Taylor N., Occhipinti P., Bridges A.A., Brangwynne C.P., Gladfelter A.S. RNA controls PolyQ protein phase transitions. Mol. Cell. 2015; 60:220–230. 26474065 10.1016/j.molcel.2015.09.017 PMC5221516

[39] Langdon E.M. , Qiu Y., Ghanbari Niaki A., McLaughlin G.A., Weidmann C.A., Gerbich T.M., Smith J.A., Crutchley J.M., Termini C.M., Weeks K.M. et al. mRNA structure determines specificity of a polyQ-driven phase separation. Science. 2018; 360:922–927. 29650703 10.1126/science.aar7432 PMC6192030

[40] O’Shaughnessy E.C. , Stone O.J., LaFosse P.K., Azoitei M.L., Tsygankov D., Heddleston J.M., Legant W.R., Wittchen E.S., Burridge K., Elston T.C. et al. Software for lattice light-sheet imaging of FRET biosensors, illustrated with a new Rap1 biosensor. J. Cell Biol. 2019; 218:3153–3160. 31444239 10.1083/jcb.201903019 PMC6719445

[41] Chen C.Y. , Chang C.ke, Chang Y.W., Sue S.C., Bai H.I., Riang L., Hsiao C.D., Huang T. huang Structure of the SARS coronavirus nucleocapsid protein RNA-binding dimerization domain suggests a mechanism for helical packaging of viral RNA. J. Mol. Biol. 2007; 368:1075–1086. 17379242 10.1016/j.jmb.2007.02.069 PMC7094638

[42] Nguyen T.H.Van , Lichière J., Canard B., Papageorgiou N., Attoumani S., Ferron F., Coutard B Structure and oligomerization state of the C-terminal region of the middle east respiratory syndrome coronavirus nucleoprotein. Acta Crystallogr. Sect. D Struct. Biol. 2019; 75:8–15. 30644840 10.1107/S2059798318014948 PMC7159728

[43] Sonn-Segev A. , Belacic K., Bodrug T., Young G., VanderLinden R.T., Schulman B.A., Schimpf J., Friedrich T., Dip P.V., Schwartz T.U. et al. Quantifying the heterogeneity of macromolecular machines by mass photometry. Nat. Commun. 2020; 11:1772. 32286308 10.1038/s41467-020-15642-w PMC7156492

[44] Tsang B. , Arsenault J., Vernon R.M., Lin H., Sonenberg N., Wang L.Y., Bah A., Forman-Kay J.D. Phosphoregulated FMRP phase separation models activity-dependent translation through bidirectional control of mRNA granule formation. Proc. Natl. Acad. Sci. U.S.A. 2019; 116:4218–4227. 30765518 10.1073/pnas.1814385116 PMC6410804

[45] Weidmann C.A. , Mustoe A.M., Jariwala P.B., Calabrese J.M., Weeks K.M. Analysis of RNA–protein networks with RNP-MaP defines functional hubs on RNA. Nat. Biotechnol. 2021; 39:347–356. 33077962 10.1038/s41587-020-0709-7 PMC7956044

[46] Smola M.J. , Rice G.M., Busan S., Siegfried N.A., Weeks K.M. Selective 2′-hydroxyl acylation analyzed by primer extension and mutational profiling (SHAPE-MaP) for direct, versatile and accurate RNA structure analysis. Nat. Protoc. 2015; 10:1643–1669. 26426499 10.1038/nprot.2015.103 PMC4900152

[47] Busan S. , Weeks K.M. Accurate detection of chemical modifications in RNA by mutational profiling (MaP) with shapemapper 2. RNA. 2018; 24:143–148. 29114018 10.1261/rna.061945.117 PMC5769742

[48] Kim D. , Lee J.Y., Yang J.S., Kim J.W., Kim V.N., Chang H. The architecture of SARS-CoV-2 transcriptome. Cell. 2020; 181:914–921. 32330414 10.1016/j.cell.2020.04.011 PMC7179501

[49] Lan T.C.T. , Allan M.F., Malsick L.E., Woo J.Z., Zhu C., Zhang F., Khandwala S., Nyeo S.S.Y., Sun Y., Guo J.U. et al. Secondary structural ensembles of the SARS-CoV-2 RNA genome in infected cells. Nat. Commun. 2022; 13:1128. 35236847 10.1038/s41467-022-28603-2 PMC8891300

[50] Kang S. , Yang M., Hong Z., Zhang L., Huang Z., Chen X., He S., Zhou Z., Zhou Z., Chen Q. et al. Crystal structure of SARS-CoV-2 nucleocapsid protein RNA binding domain reveals potential unique drug targeting sites. Acta Pharm. Sin. B. 2020; 10:1228–1238. 32363136 10.1016/j.apsb.2020.04.009 PMC7194921

[51] Zinzula L. , Basquin J., Bohn S., Beck F., Klumpe S., Pfeifer G., Nagy I., Bracher A., Hartl F.U., Baumeister W. High-resolution structure and biophysical characterization of the nucleocapsid phosphoprotein dimerization domain from the Covid-19 severe acute respiratory syndrome coronavirus 2. Biochem. Biophys. Res. Commun. 2021; 538:54–62. 33039147 10.1016/j.bbrc.2020.09.131 PMC7532810

[52] Yuan S. , Peng L., Park J.J., Hu Y., Devarkar S.C., Dong M.B., Shen Q., Wu S., Chen S., Lomakin I.B. et al. Nonstructural protein 1 of SARS-CoV-2 is a potent pathogenicity factor redirecting host protein synthesis machinery toward viral RNA. Mol. Cell. 2020; 80:1055–1066. 33188728 10.1016/j.molcel.2020.10.034 PMC7833686

[53] Tidu A. , Janvier A., Schaeffer L., Sosnowski P., Kuhn L., Hammann P., Westhof E., Eriani G., Martin F. The viral protein NSP1 acts as a ribosome gatekeeper for shutting down host translation and fostering SARS-CoV-2 translation. RNA. 2021; 27:253–264. 10.1261/rna.078121.120 PMC7901841 33268501

[54] Zeng W. , Liu G., Ma H., Zhao D., Yang Y., Liu M., Mohammed A., Zhao C., Yang Y., Xie J. et al. Biochemical characterization of SARS-CoV-2 nucleocapsid protein. Biochem. Biophys. Res. Commun. 2020; 527:618–623. 32416961 10.1016/j.bbrc.2020.04.136 PMC7190499

[55] Zhao H. , Wu D., Nguyen A., Li Y., Adão R.C., Valkov E., Patterson G.H., Piszczek G., Schuck P. Energetic and structural features of SARS-CoV-2 N-protein co-assemblies with nucleic acids. Iscience. 2021; 24:102523. 33997662 10.1016/j.isci.2021.102523 PMC8103780

[56] Sun L. , Li P., Ju X., Rao J., Huang W., Ren L., Zhang S., Xiong T., Xu K., Zhou X. et al. In vivo structural characterization of the SARS-CoV-2 RNA genome identifies host proteins vulnerable to repurposed drugs. Cell. 2021; 184:1865–1883. 33636127 10.1016/j.cell.2021.02.008 PMC7871767

[57] Huston N.C. , Wan H., Strine M.S., de Cesaris Araujo Tavares R., Wilen C.B., Pyle A.M. Comprehensive in vivo secondary structure of the SARS-CoV-2 genome reveals novel regulatory motifs and mechanisms. Mol. Cell. 2021; 81:584–598. 33444546 10.1016/j.molcel.2020.12.041 PMC7775661

[58] Kim T.H. , Tsang B., Vernon R.M., Sonenberg N., Kay L.E., Forman-Kay J.D. Phospho-dependent phase separation of FMRP and CAPRIN1 recapitulates regulation of translation and deadenylation. Science. 2019; 365:825–829. 31439799 10.1126/science.aax4240

[59] Grossoehme N.E. , Li L., Keane S.C., Liu P., Dann C.E., Leibowitz J.L., Giedroc D.P. Coronavirus N protein N-Terminal domain (NTD) specifically binds the transcriptional regulatory sequence (TRS) and melts TRS-cTRS RNA duplexes. J. Mol. Biol. 2009; 394:544–557. 19782089 10.1016/j.jmb.2009.09.040 PMC2783395

[60] Caruso Í.P. , Sanches K., Da Poian A.T., Pinheiro A.S., Almeida F.C.L. Dynamics of the SARS-CoV-2 nucleoprotein N-terminal domain triggers RNA duplex destabilization. Biophys. J. 2021; 120:2814–2827. 34197802 10.1016/j.bpj.2021.06.003 PMC8239202

[61] Mebus-antunes C. , Neves-martins T.C., De Sá J.M. Structure insights, thermodynamic profiles, dsDNA melting activity, and liquid- liquid phase separation of the SARS-CoV-2 nucleocapsid N-terminal domain binding to DNA. 2021;

[62] Enjuanes L. , Almazán F., Sola I., Zuñiga S. Biochemical aspects of coronavirus replication and virus-host interaction. Annu. Rev. Microbiol. 2006; 60:211–230. 16712436 10.1146/annurev.micro.60.080805.142157

[63] Krainer G. , Welsh T.J., Joseph J.A., Espinosa J.R., Wittmann S., de Csilléry E., Sridhar A., Toprakcioglu Z., Gudiškytė G., Czekalska M.A. et al. Reentrant liquid condensate phase of proteins is stabilized by hydrophobic and non-ionic interactions. Nat. Commun. 2021; 12:1085. 33597515 10.1038/s41467-021-21181-9 PMC7889641

[64] Bar-On Y.M. , Flamholz A., Phillips R., Milo R. SARS-CoV-2 (COVID-19) by the numbers. Elife. 2020; 9:e57309. 32228860 10.7554/eLife.57309 PMC7224694

[65] Yao H. , Song Y., Chen Y., Wu N., Xu J., Sun C., Zhang J., Weng T., Zhang Z., Wu Z. et al. Molecular architecture of the SARS-CoV-2 virus. Cell. 2020; 183:730–738. 32979942 10.1016/j.cell.2020.09.018 PMC7474903

[66] Klein S. , Cortese M., Winter S.L., Wachsmuth-Melm M., Neufeldt C.J., Cerikan B., Stanifer M.L., Boulant S., Bartenschlager R., Chlanda P. SARS-CoV-2 structure and replication characterized by in situ cryo-electron tomography. Nat. Commun. 2020; 11:5885. 33208793 10.1038/s41467-020-19619-7 PMC7676268

[67] Jawerth L. , Fischer-Friedrich E., Saha S., Wang J., Franzmann T., Zhang X., Sachweh J., Ruer M., Ijavi M., Saha S. et al. Protein condensates as aging Maxwell fluids. Science (80-.). 2020; 370:1317–1323. 10.1126/science.aaw4951 33303613

[68] Raman S. , Brian D.A. Stem-Loop IV in the 5′ untranslated region is a cis-Acting element in bovine coronavirus defective interfering RNA replication. J. Virol. 2005; 79:12434–12446. 16160171 10.1128/JVI.79.19.12434-12446.2005 PMC1211515

[69] Yang D. , Liu P., Giedroc D.P., Leibowitz J. Mouse hepatitis virus stem-loop 4 functions as a spacer element required to drive subgenomic RNA synthesis. J. Virol. 2011; 85:9199–9209. 21715502 10.1128/JVI.05092-11 PMC3165806

[70] Kang H. , Feng M., Schroeder M.E., Giedroc D.P., Leibowitz J.L. Putative cis-Acting stem-loops in the 5′ untranslated region of the severe acute respiratory syndrome coronavirus can substitute for their mouse hepatitis virus counterparts. J. Virol. 2006; 80:10600–10614. 16920822 10.1128/JVI.00455-06 PMC1641749

[71] Guan B.-J. , Wu H.-Y., Brian D.A. An optimal cis-Replication stem-loop IV in the 5′ untranslated region of the mouse coronavirus genome extends 16 nucleotides into open reading frame 1. J. Virol. 2011; 85:5593–5605. 21430057 10.1128/JVI.00263-11 PMC3094970

[72] Kang H. , Feng M., Schroeder M.E., Giedroc D.P., Leibowitz J.L. Stem-loop 1 in the 5′ UTR of the SARS coronavirus can substitute for its counterpart in mouse hepatitis virus. Adv. Exp. Med. Biol. 2006; 581:105–108. 17037514 10.1007/978-0-387-33012-9_18 PMC7123683

[73] Takeda M. , Chang C.ke, Ikeya T., Güntert P., Chang Y. hsiang, Hsu Y. lan, Huang T. huang, Kainosho M. Solution structure of the C-terminal dimerization domain of SARS coronavirus nucleocapsid protein solved by the SAIL-NMR method. J. Mol. Biol. 2008; 380:608–622. 18561946 10.1016/j.jmb.2007.11.093 PMC7094413

[74] Yang D. , Leibowitz J.L. The structure and functions of coronavirus genomic 3′ and 5′ ends. Virus Res. 2015; 206:120–133. 25736566 10.1016/j.virusres.2015.02.025 PMC4476908

[75] Fung T.S. , Liu D.X. Post-translational modifications of coronavirus proteins: roles and function. Future Virol. 2018; 13:405–430. 32201497 10.2217/fvl-2018-0008 PMC7080180

[76] Wu C.H. , Yeh S.H., Tsay Y.G., Shieh Y.H., Kao C.L., Chen Y.S., Wang S.H., Kuo T.J., Chen D.S., Chen P.J. Glycogen synthase kinase-3 regulates the phosphorylation of severe acute respiratory syndrome coronavirus mucleocapsid protein and viral replication. J. Biol. Chem. 2009; 284:5229–5239. 19106108 10.1074/jbc.M805747200 PMC8011290

[77] Syed A.M. , Taha T.Y., Khalid M.M., Tabata T., Chen I.P., Sreekumar B., Chen P.-Y., Hayashi J.M., Soczek K.M., Ott M. et al. Rapid assessment of SARS-CoV-2 evolved variants using virus-like particles. 2021; bioRxiv doi:05 August 2021, preprint: not peer reviewed 10.1101/2021.08.05.455082. PMC9005165 34735219

[78] Yaron T.M. , Heaton B.E., Levy T.M., Johnson J.L., Jordan T.X., Cohen B.M., Kerelsky A., Lin T.-Y., Liberatore K.M., Bulaon D.K. et al. The FDA-approved drug Alectinib compromises SARS-CoV-2 nucleocapsid phosphorylation and inhibits viral infection in vitro. 2020; bioRxiv doi:16 December 2020, preprint: not peer reviewed 10.1101/2020.08.14.251207.

[79] Li L. , Kang H., Liu P., Makkinje N., Williamson S.T., Leibowitz J.L., Giedroc D.P. Structural lability in stem-loop 1 drives a 5′ UTR-3′ UTR interaction in coronavirus replication. J. Mol. Biol. 2008; 377:790–803. 18289557 10.1016/j.jmb.2008.01.068 PMC2652258

[80] Syed A.M. , Taha T.Y., Tabata T., Chen I.P., Ciling A., Khalid M.M., Sreekumar B., Chen P.Y., Hayashi J.M., Soczek K.M. et al. Rapid assessment of SARS-CoV-2–evolved variants using virus-like particles. Science. 2021; 374:1626–1632. 34735219 10.1126/science.abl6184 PMC9005165

[81] Cao C. , Cai Z., Xiao X., Rao J., Chen J., Hu N., Yang M., Xing X., Wang Y., Li M. et al. The architecture of the SARS-CoV-2 RNA genome inside virion. Nat. Commun. 2021; 12:3917. 34168138 10.1038/s41467-021-22785-x PMC8225788

[82] Seim I. , Roden C.A., Gladfelter A.S. Role of spatial patterning of N-protein interactions in SARS-CoV-2 genome packaging. Biophys J. 2021; 120:2771–2784. 34214535 10.1016/j.bpj.2021.06.018 PMC8241574

[83] Finkel Y. , Mizrahi O., Nachshon A., Weingarten-Gabbay S., Morgenstern D., Yahalom-Ronen Y., Tamir H., Achdout H., Stein D., Israeli O. et al. The coding capacity of SARS-CoV-2. Nature. 2021; 589:125–130. 32906143 10.1038/s41586-020-2739-1

[84] Ma W. , Zheng G., Xie W., Mayr C. In vivo reconstitution finds multivalent RNA–RNA interactions as drivers of mesh-like condensates. Elife. 2021; 10:e64252. 33650968 10.7554/eLife.64252 PMC7968931

