# Supplementary material for: Double-stranded RNA drives SARS-CoV-2 nucleocapsid protein to undergo phase separation at specific temperatures
Source: Nucleic Acids Res. 2022 Jul 25;50(14):8168–92. doi: 10.1093/nar/gkac596 (PMC9371935; doi:10.1093/nar/gkac596)
Supplement: gkac596_Supplemental_Files [file gkac596_supplemental_files.zip › Roden_Supplemental_Figures_Revision.pdf]

Fig. S1 dsRNA-driven LLPS is independent of RBD1

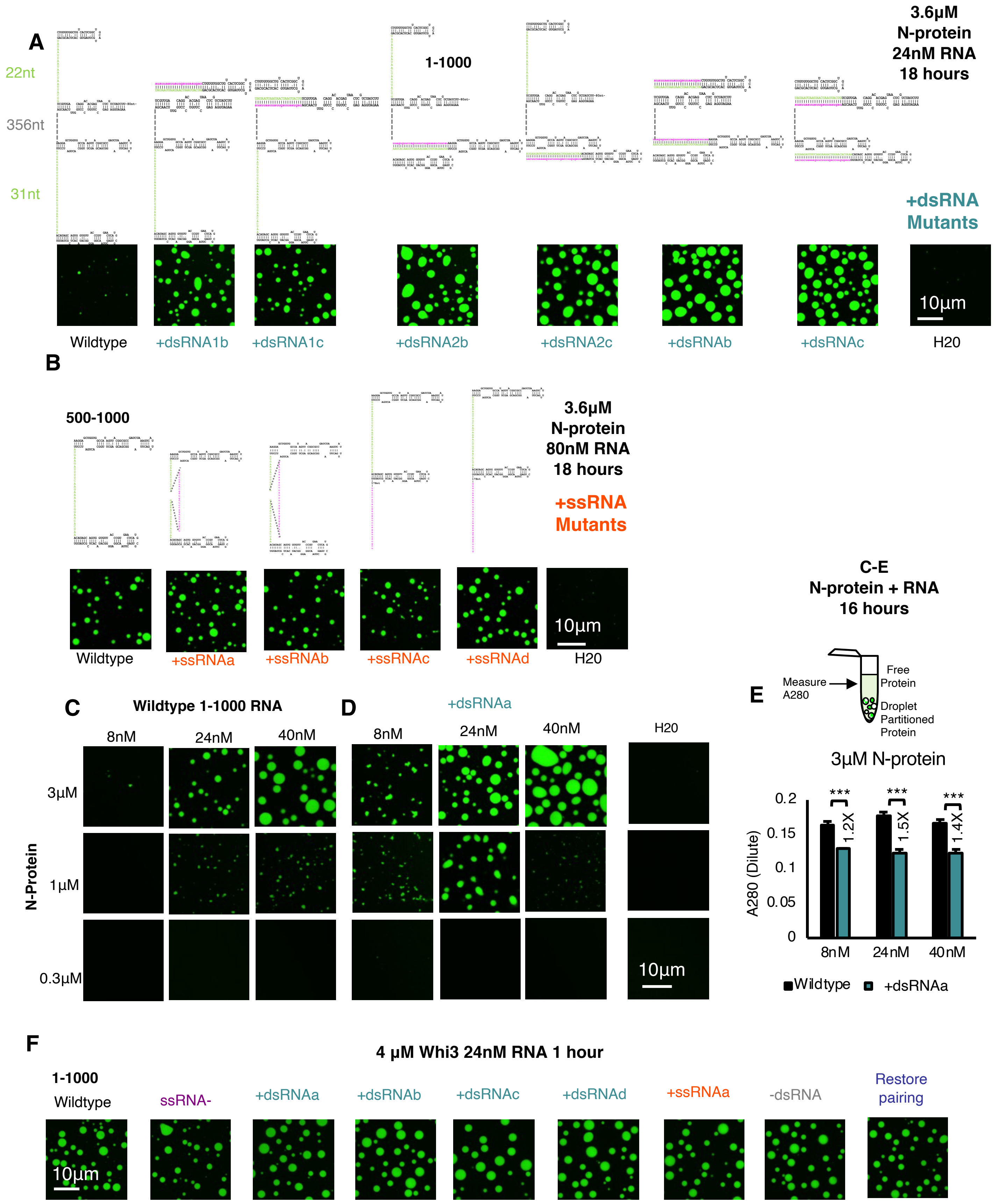

(A) At 3.6 µM N-protein (green) and 24nM RNA following 18 hours of incubation, mutations in the context of 1-1000 nt which increase the dsRNA (+dsRNA teal) of principal site 1 only, 2 only, or 1, and 2 lead to enhanced condensation in comparison to wildtype regardless of whether the anti-sense sequence to the principal site was inserted on the 5′ or 3′ side. Enhancement of condensation may be length dependent. H2O indicates equivalent volume of added water only control. **(B)** Location or sequence of ssRNA (+ssRNA orange) insertion leads to equally negligible levels of condensation enhancement. 3.6 µM N-protein, 80 nM RNA 18 hours of incubation in the 500-1000 sequence context. **(C-E)** Phase diagrams for equivalent RNA and N-protein concentrations (3 µM, 1 µM, 0.3 µM) for 1-1000 wildtype, +dsRNAa for 8 nM, 24 nM, or 40 nM RNA. At 3 µM, +dsRNAa leads to more condensation relative to Wild-type 1-1000 for all tested RNA concentrations. At 1 µM +dsRNAa shifts the phase boundary to the left relative to wildtype. 0.3 µM N-protein does not drive condensation for any sequence at any tested RNA concentration. **(E)** A280 absorbance of for 3 µM N-protein concentration for panels **S1C, D**. For all tested RNA concentrations relative to wildtype, +dsRNAa has less protein in solution following 16 hours incubation as measured by A280 nanodrop. Error bars mark standard deviation for the three replicates and * indicate significance, students T test (*** $p<0.001$, ** $p<0.01$, ns not significant) with brackets showing comparison for the indicated statistical test. **(F)** 1-1000 RNA and its mutants (purple -ssRNA, teal +dsRNA, oranges -ssRNA, -dsRNA gray, Restore pairing blue) lead to equivalent condensation of Whi3 protein (green). For all images scale bar indicates 10 µm. All experiments show representative images from at least 3 replicates.

Fig. S2: RNA structure mutants accelerate droplet formation cell free and in cells

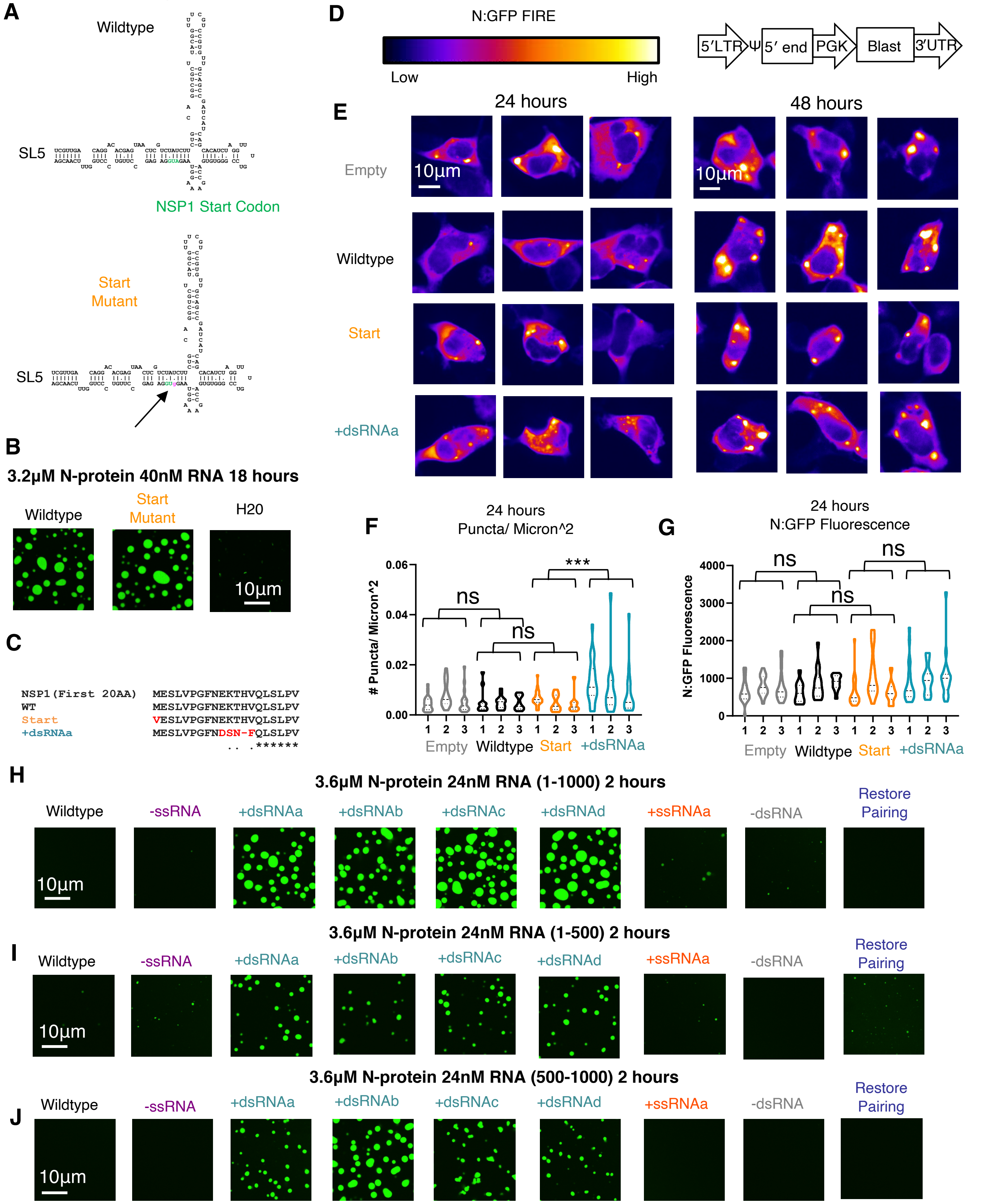

(A) Structure model of SL5 for SARS-CoV-2 with the location of the start codon of non-structural protein 1 (NSP1) in orange text. AU base pair of the start codon is replaced with a GU wobble pair (ATG --> gTG) to eliminate NSP1 translation while sparing RNA structure. **(B)** Wildtype and Start Mutant RNA result in similar levels of condensation cell free. 3.2µM N-protein (green) and 40nM RNA following 2.5 hours of incubation. **(C)** NSP1 protein sequence of mutants tested in **S2E-G** destroy NSP1 production. **(D)** N: GFP protein signal (FIRE blue low signal white high signal) key and 5′end overexpression plasmids design. **(E)** Representative HEK293T cells co-transfected with N: GFP and the indicated 5′end fragment at 24 hours (left 3 panels) or 48 hours (right 3 panels). +dsRNAa mutant produces more puncta at 24 hours (4-5 per cell) compared to wildtype, start, or empty (2-3 per cell). Difference is reduced at 48 hours (4-5 puncta in all three 5′end containing cells). **(F)** Quantification of the number of puncta per Micron^2 at 24 hours. +dsRNAa produces significantly more puncta per unit area then the Start mutant. * indicate significance students T test (*** $p<0.001$, ns not significant) with brackets showing comparison for the indicated statistical test. **(G)** Quantification of the mean intensity of N: GFP signal at 24 hours. Analyzed cells have similar GFP signal distribution. No comparisons are significant (ns). **(H)** Addition of dsRNA (teal) (+dsRNAa-d) enhances N-protein condensation. Representative images from the two-hour incubation timepoint for panels **1F**. N-protein signal is shown in green. **(I and J)** Representative images from the two-hour incubation timepoint for panels **Figure 1D** (1-500) and **1E** (500-1000). For all images scale bar indicates 10µm.

Fig. S3: Addition of dsRNA alters material properties

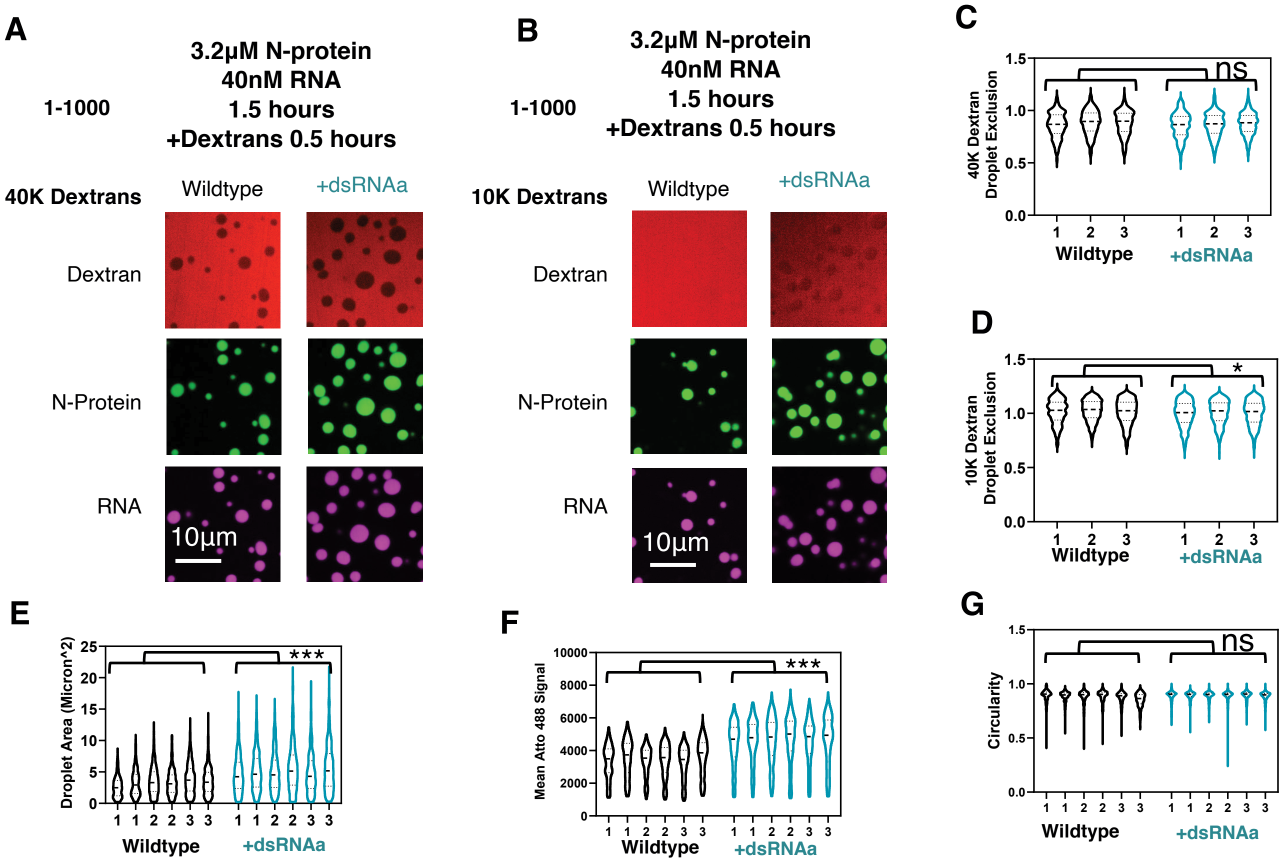

(A) 40K dextrans are excluded from N-protein condensates made with wildtype 1-1000 or +dsRNAa, **(B)** N-protein condensates made with wildtype 1-1000, +dsRNAa, are at least partially permeable to 10K dextrans. **(A and B)** Dextrans are shown in red, N-protein is shown in green, RNA in purple. 3.2 μM N-protein 40 nM RNA 1.5 hours incubation followed by 0.5 hours of incubation with dextrans. For all images scale bar indicates 10μm all experiments show representative images from 3 replicates. **(C-G)** Quantification of A and B. **(C)** Relative Dextran exclusion (droplet signal divide by non-droplet signal) for N-protein condensates made with wildtype 1-1000 or +dsRNAa, all tested RNAs equivalently exclude 40K Dextrans. **(D)** 10K Dextrans are weakly but significantly excluded from +dsRNAa. Plotted is the relative Dextran exclusion (droplet signal divide by non-droplet signal) for N-protein condensates made with wildtype 1-1000 or +dsRNAa. **(E)** +dsRNAa droplets are significantly larger (N-protein Atto288 signal area) than droplets formed with wildtype RNA. **(F)** +dsRNAa droplets have significantly more Atto488 fluorescence (N-protein label) then wildtype indicative of more protein recruitment. **(G)** No difference in circularity between wildtype and +dsRNAa droplets. For **S3C-G,** * indicates significance students T test (*** $p<0.001$, **$p<0.01$, *$p<0.05$, ns not significant) with brackets showing comparison.

Fig. S4 RNA sequence and structure encodes N-protein LCST Behavior via RBD2

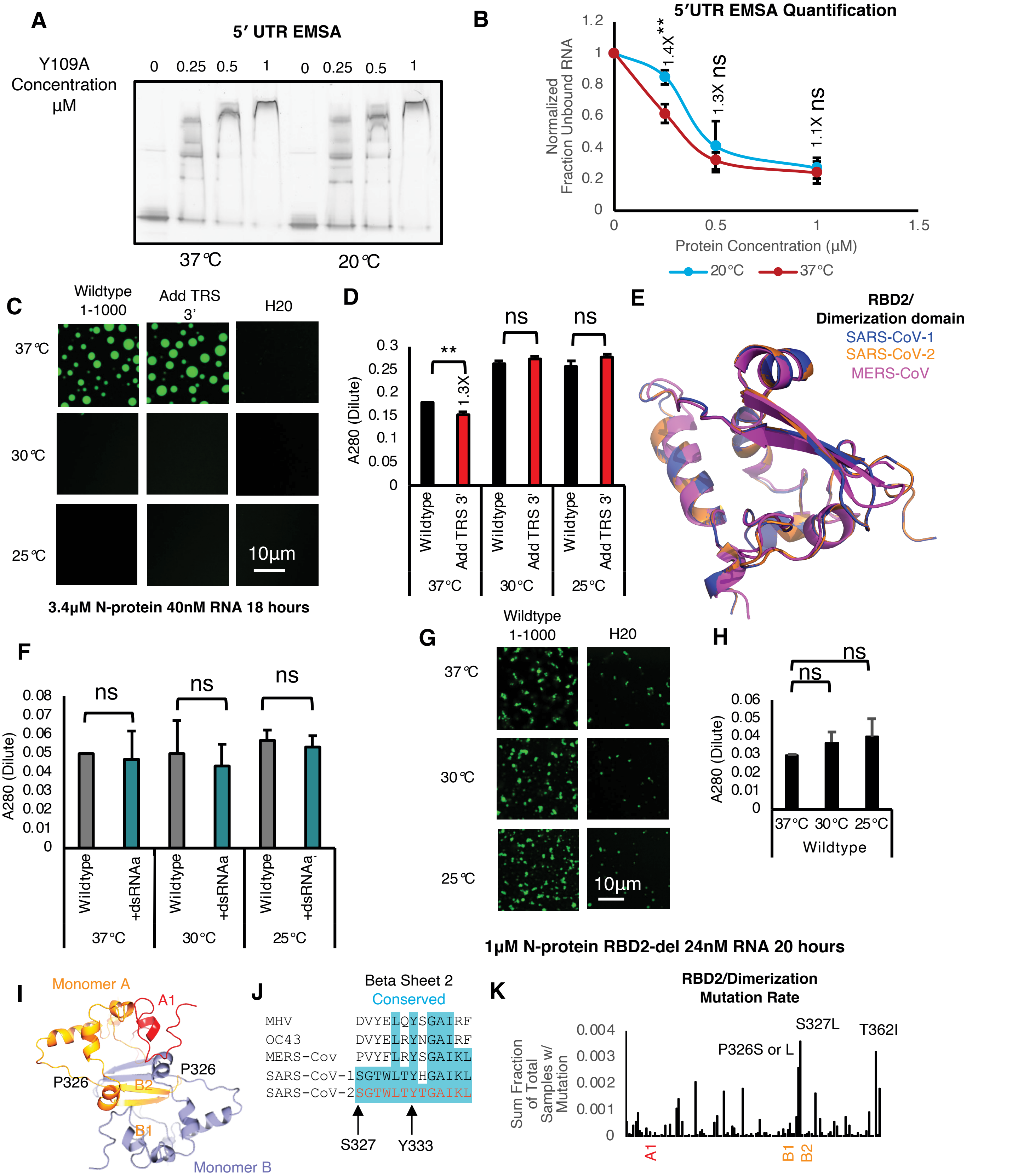

(A) Representative EMSA for Y109A mutant N-protein and 5′UTR RNA at 37 and 25°C. **(B)** EMSA Quantification; 3 replicates. Less unbound 5′UTR RNA at 37°C incubation then 25°C incubation. Y axis is the normalized fraction unbound protein (0µM protein condition set to 1). X axis tested Y109A protein concentration. 37°C red line, 25°C, blue line error bars reflect the standard deviation. **(C)** Addition of a second TRS sequence structure motif (RBD1 binding site) does not alter the LCST behavior of N-protein. Wildtype 1-1000 5′end RNA or Add TRS 3′ mutant was incubated at 37°C, 30°C or 25°C for a period of 18 hours (N-protein signal shown in green). **(D)** A280 measurement of remaining N-protein in the solution for **Fig. S4C**. Only 37°C shows a difference in signal consistent with previous results **(Fig. 2B)**. **(E)** Alignment of the predicted structure for SARS-CoV-2 (orange ribbon), the crystal structure of MERS-CoV (magenta ribbon), to the crystal structure of SARS-CoV-1 (blue ribbon) RBD2 dimerization domain. Proteins may adopt similar folds. **(F)** A280 measurement of remaining N-protein in the solution for **Fig. 2G**. **(G)** Repeat of LCST experiment with wildtype RNA at lower N-protein (1µM) and RNA (15nM) concentration. H20 alone results in less condensation indicative for some RNA dependence for N-protein RBD-2 Del. **(H)** A280 measurements for **(Fig. S4G)** At 37°C, 30°C and 25°C wildtype RNA has not significantly (ns) different A280 measurements indicative of similar amounts of protein in solution and identical amounts of condensation. For all A280 measurements, error bars mark standard deviation for the three replicates and brackets indicate the comparison for students T test (** p<0.01,ns not significant). For all images scale bar indicates 10µm. All experiments show representative images from at least 3 replicates. **(I)** Structure model of the RBD2 dimerization domain (depicted as two monomers to highlight dimerization interface, beta sheet 2) for SARS-CoV-2 (red sequences/ red ribbon) indicate the location of the RBD2. **(J)** Primary sequence conservation beta sheet 2 of the dimerization interface. **(K)** Fraction of patient samples with mutations in the indicated amino acids of RBD2/Dimerization domain.

Fig. S5: RBD1 dependent crosslinking adjacent to YYAAAY motif; distributed throughout the genome.

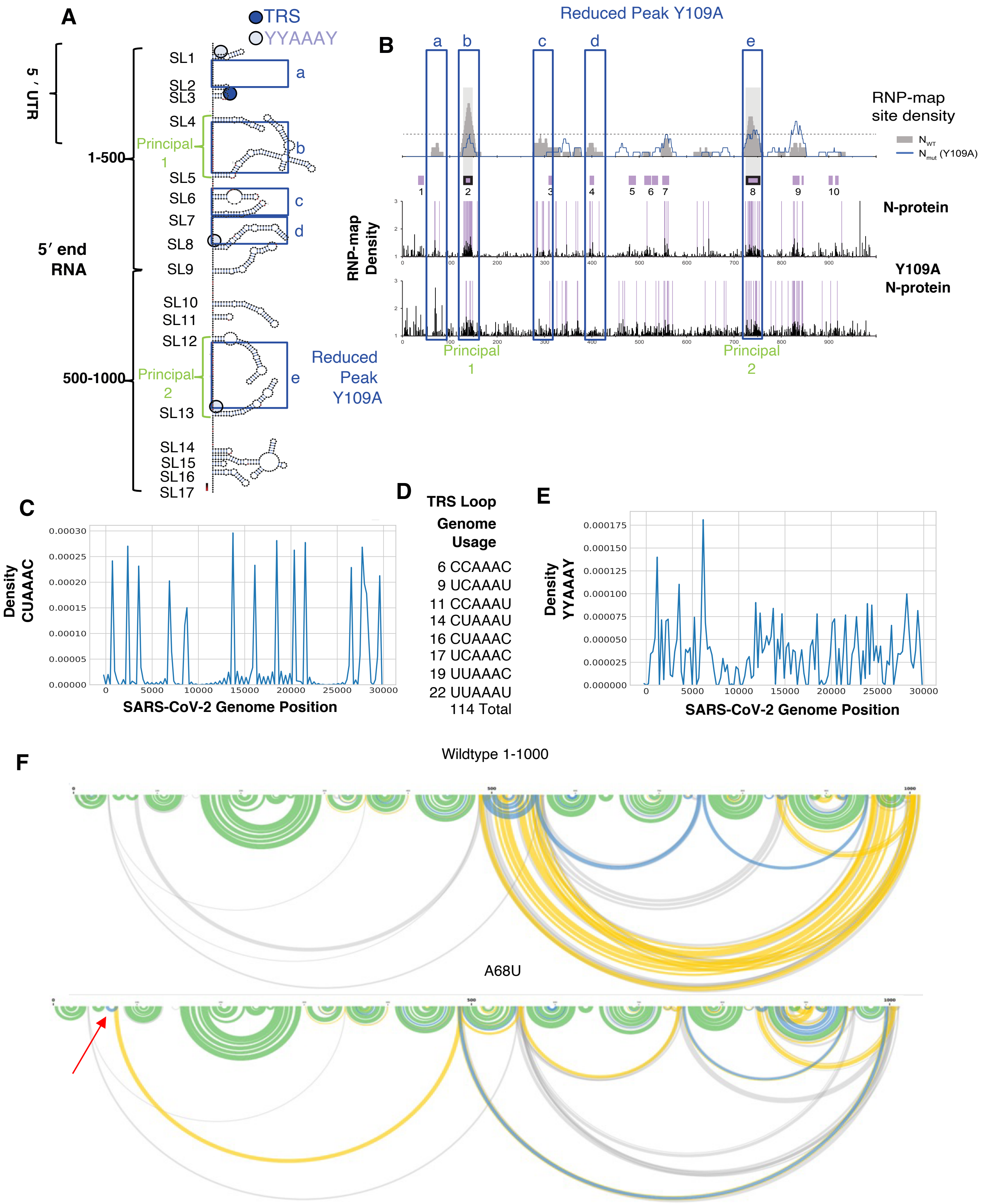

(A) Structure of the 5′end 1-1000 RNA adapted from Iserman et al. (Iserman et al., 2020). Blue squares indicate the location of reduced N-protein crosslinking following Y109A mutation which destroys RBD1 (see **B**). Dark Blue and light blue circles indicate locations of the perfect TRS loop sequence (CUAAAC dark blue) verses (YYAAAY light blue) showing that peak reduction is often adjacent to TRS or TRS-like motifs. **(C) (C-E)** Density of TRS-Loop-like motif (YYAAAY) across the genome other than CUAAAC. Y axis is the relative density of the indicated sequence across the SARS-CoV-2 genome. **(F)** SHAPE arc plots for Wildtype and A68U RNA sequences.

Fig. S6: RNA sequence/ structure elements encode N-protein genome interactions

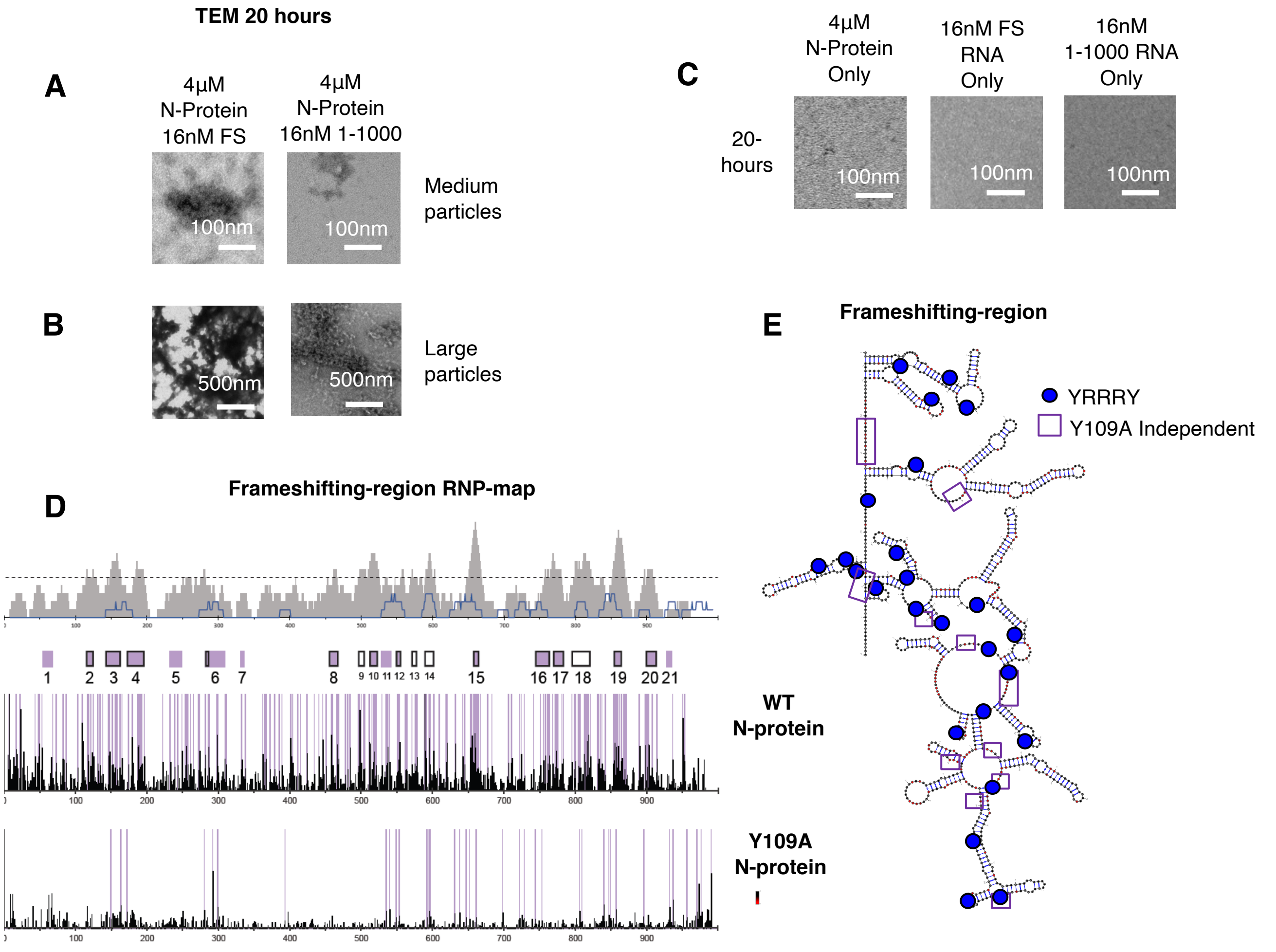

(A and B) Representative TEM images from the 20-hour timepoint depicting medium sized **(A)** and large sized **(B)** clusters of N-protein and either 10nM frameshift left panels or 1-1000 5′end RNA. Larger clusters were not detected at the 20-minute timepoint. **(A)** Scale bar is 100nm. **(B)** Scale bar is 500nm. **(C)** RNA or N-protein alone does not form particles following 20-hours of incubation at room temperature. Representative TEM images of 4μ M N-protein, 16nM FS RNA, or 16nM 1-1000 5′end incubated for 20-hours at room temperature. Scale bar is 100nM. **(D and E)** RNP-map data for FS RNA with either wildtype or Y109A mutant N-protein. Majority of crosslinking is lost following RBD1 mutation. FS contains YRRRY motifs (dark blue circles). Peaks retained following Y109A mutation (purple squares) are in the vicinity of structured RNA and largely do not overlap with YRRRY motifs.
